# Iron Metabolism and Idiopathic Pulmonary Arterial Hypertension: New Insights from Bioinformatic Analysis

**DOI:** 10.1155/2021/5669412

**Published:** 2021-10-22

**Authors:** Hua-Xi Zou, Bai-Quan Qiu, Song-Qing Lai, Xue-Liang Zhou, Cheng-Wu Gong, Li-Jun Wang, Ming-Ming Yuan, An-Di He, Ji-Chun Liu, Huang Huang

**Affiliations:** ^1^Department of Cardiothoracic Surgery, First Affiliated Hospital, Nanchang University, Nanchang, Jiangxi 330006, China; ^2^Institute of Cardiovascular Diseases, Jiangxi Academy of Clinical Medical Sciences, The First Affiliated Hospital of Nanchang University, Nanchang 330006, China; ^3^Department of Cardiothoracic Surgery, Second Affiliated Hospital, Nanchang University, Nanchang, Jiangxi 330006, China

## Abstract

Idiopathic pulmonary arterial hypertension (IPAH) is a rare vascular disease with a poor prognosis, and the mechanism of its development remains unclear. Further molecular pathology studies may contribute to a comprehensive understanding of IPAH and provide new insights into diagnostic markers and potential therapeutic targets. Iron deficiency has been reported in 43-63% of patients with IPAH and is associated with reduced exercise capacity and higher mortality, suggesting that dysregulated iron metabolism may play an unrecognized role in influencing the development of IPAH. In this study, we explored the regulatory mechanisms of iron metabolism in IPAH by bioinformatic analysis. The molecular function of iron metabolism-related genes (IMRGs) is mainly enriched in active transmembrane transporter activity, and they mainly affect the biological process of response to oxidative stress. Ferroptosis and fluid shear stress and atherosclerosis pathways may be the critical pathways regulating iron metabolism in IPAH. We further identified 7 key genes (BCL2, GCLM, MSMO1, SLC7A11, SRXN1, TSPAN5, and TXNRD1) and 5 of the key genes (BCL2, MSMO1, SLC7A11, TSPAN5, and TXNRD1) as target genes may be regulated by 6 dysregulated miRNAs (miR-483-5p, miR-27a-3p, miR-27b-3p, miR-26b-5p, miR-199a-5p, and miR-23b-3p) in IPAH. In addition, we predicted potential IPAH drugs—celastrol and cinnamaldehyde—that target iron metabolism based on our results. These results provide insights for further definition of the role of dysregulated iron metabolism in IPAH and contribute to a deeper understanding of the molecular mechanisms and potential therapeutic targets of IPAH.

## 1. Introduction

Pulmonary arterial hypertension (PAH) is a rare vascular disease with high morbidity and mortality, characterized by pulmonary vascular remodeling and increased pulmonary vascular resistance, ultimately resulting in right ventricular failure and death [1, 2]. There are significant differences in the progression and prognosis between patients with PAH of different etiologies, ethnicities, and genetic mutations, suggesting that targeted therapies are necessary to improve the overall prognosis of patients [3–5].

Idiopathic PAH (IPAH) is a specific type of PAH without any family history of PAH or known pathogenic factors, and patients with IPAH tend to show worse survival compared to PAH associated with congenital heart disease [4, 6]. Although the pulmonary hemodynamics, exercise capacity, and life quality of IPAH patients have improved considerably with advances in diagnosis and treatment, there is still no satisfactory cure available [6–8]. An essential understanding of the molecular and pathological mechanism may provide new insights for the therapy for IPAH.

Iron is an essential element in basic biological processes, which contributes to a multitude of crucial physiologic processes [9]. Iron deficiency has been reported in 43-63% of patients with IPAH and is associated with reduced exercise capacity and higher mortality [10–12]. Studies have shown that intracellular iron deficiency in pulmonary arterial smooth muscle cells could alter pulmonary vascular function, and rats on an iron-deficient diet exhibit significant pulmonary vascular remodeling with prominent muscularization, medial hypertrophy, and perivascular inflammatory cell infiltration, associated with elevated pulmonary artery pressure and right ventricular hypertrophy [13, 14], which indicates iron metabolism participating in the maintenance of pulmonary vascular homeostasis, and dysregulated iron metabolism may play an important role in the development of IPAH.

Since current animal models provide little accurate information on the pathobiology of human IPAH and the value of developing and validating drug therapy is debatable, the research on human specimens should be paid more attention [6, 15]. The advancement of gene microarray expression analysis has greatly contributed to the exploration of crucial genes in the pathobiology of IPAH [16, 17], and microarray datasets from lung tissue may provide a more accurate and direct reflection of the pathobiology of IPAH than peripheral blood. Here, to determine the role of iron metabolism in IPAH, we identified iron metabolism-related genes (IMRGs) based on relevant databases and analyzed the differential expression of IMRGs among IPAH and normal samples in the microarray dataset GSE117261. Gene Ontology (GO) and Kyoto Encyclopedia of Genes and Genomes (KEGG) analyses of differentially expressed IMRGs (DEIMRGs) were further performed, and protein-protein interaction (PPI) network was constructed to identify key modules and hub genes. Studies have demonstrated that microRNAs (miRNAs) exert an essential effect on IPAH by negatively regulating target mRNA [15]; we constructed a miRNA-mRNA network to explore the potential regulation of IMRG by miRNAs in IPAH. The expression and diagnostic value of hub genes and target DEIMRGs (tIMRG) were further validated in the microarray dataset GSE15197 to identify key genes and crucial miRNA-IMRG networks. Finally, we predicted potential therapeutic drugs for IPAH based on our findings.

## 2. Materials and Methods

### 2.1. Data Collection

The microarray datasets GSE117261 and GSE15197 were downloaded from the Gene Expression Omnibus (GEO) database (https://www.ncbi.nlm.nih.gov/geo/). The dataset GSE117261 contains whole transcriptome expression data from 32 IPAH lung samples and 25 normal lung samples, used to screen for DEIMRGs. The dataset GSE15197 contains whole transcriptome expression data from 18 IPAH lung samples and 13 normal lung samples, used to verify the target DEIMRGs and hub genes. After ID conversion, the average expression value was taken as the gene expression value when multiple probes correspond to one gene. The raw data were log2 transformed and quantile normalized before analyses.

### 2.2. Identification of DEGs and DEIMRGs

DEGs were identified using the limma package (version 3.48.0) in R software (version 4.1), *p* values were adjusted using the Benjamini and Hochberg method [18]. The false discovery rate (FDR) < 0.05 and ∣log2FC | ≥0.25 were defined as the selection thresholds for selecting the DEGs.

IMRGs were identified from related gene sets (GOBP_IRON_ION_HOMEOSTASIS, GOBP_IRON_ION_TRANSPORT, GOBP_HEME_METABOLIC_PROCESS, GOBP_RESPONSE_TO_IRON_ION, GOBP_IRON_SULFUR_CLUSTER_ASSEMBLY, HALLMARK_HEME_METABOLISM, GOMF_IRON_ION_BINDING, REACTOME_IRON_UPTAKE_AND_TRANSPORT, and HP_ABNORMALITY_OF_IRON_HOMEOSTASIS) in the MSigDb database [19] (https://www.gsea-msigdb.org/gsea/msigdb/index.jsp) and ferroptosis-related genes in the FerrDb database [20] (http://www.zhounan.org/ferrdb). After deduplication of genes, the merged IMRG set contains 710 genes, listed in Table S1. Finally, 88 overlapped DEIMRGs were selected using the VennDiagram package (1.6.20) in R software.

### 2.3. GO and KEGG Enrichment Analyses

The DEIMRGs identified were subjected to GO and KEGG enrichment analysis. The clusterProfiler package (version 3.12.0) was used in R software to perform the GO and KEGG enrichment analyses. The results with FDR < 0.05 were considered significantly enriched by DEIMRGs.

### 2.4. Construction of DRmiRNA–DEIMRG Regulatory Network

Dysregulated miRNAs (DRmiRNAs) in IPAH were extracted from previous studies. We searched the literature related to miRNAs and IPAH in the PubMed database (https://pubmed.ncbi.nlm.nih.gov/) [21] and excluding nonhuman specimen studies and studies without validation; a total of 23 DRmiRNAs were identified, 14 of which were upregulated and 9 downregulated, and the source studies and validation methods for each DRmiRNA are detailed in Table S2. The mirDIP database (http://ophid.utoronto.ca/mirDIP/), an integrative database of human microRNA target predictions [22], was used to predict the target mRNAs of DRmiRNAs; target mRNAs with very high score class were selected. To visualize the relationship between DRmiRNAs and predicted target mRNAs, we built a miRNA-mRNA network using Cytoscape (version 3.8.2). tIMRGs were identified using the VennDiagram package (1.6.20) in R software.

### 2.5. PPI Network Construction and Identification of Key Modules and Hub Genes

The STRING database (https://string-db.org/) and Cytoscape software were used to construct a PPI network; PPI network of DEIMRGs was constructed using the STRING database and visualized in Cytoscape software. Three functional modules were identified by the Cytoscape plugin MCODE (the parameters were set to default: degree cutoff = 2, node score cutoff = 0.2, *K* − core = 3, and Max depth = 100). Another plugin, Cytohubba, was used to identify hub genes. The built-in MCC algorithm of Cytohubba assigned a value to each gene in the PPI network and ranked these genes by values; the top 10 genes were significant and regarded as hub genes.

### 2.6. Validation of Hub Genes and tIMRGs in GSE15197

The microarray dataset GSE15197 was used for validation. After data preprocessing as described previously, the expression data of hub genes and tIMRGs were extracted and groups were compared using the *t*-test; the results with *p* < 0.05 were considered statistically significant. Receiver operating characteristic (ROC) curve analyses were performed using the HiPlot software (version 0.1.0) to determine sensitivity and specificity of hub genes and tIMRGs; the multiple gene ROC analysis was performed based on the predictive probability of multiple genes for the outcome in each sample calculated by binary logistic regression using SPSS version 22.0. Results were quantified by the area under the ROC curve (AUC); genes with AUC > 0.6 were considered to have diagnostic value.

### 2.7. Immune Infiltration Analyses

To estimate the proportion of infiltrating immune cells, normalized gene expression data of GSE117261 and GSE15197 were submitted to HiPlot software (version 0.1.0). The proportion of infiltrating immune cells was calculated with the CIBERSORT algorithm, *t*-test was used for comparison between groups, and linear regression analysis was used to analyze the correlation between gene expression and the proportion of immune cells. The results with *p* < 0.05 were defined as a statistically significant difference.

### 2.8. Potential Therapeutic Drug Prediction

We used protein-drug interaction data from the DSigDB database [23] (http://tanlab.ucdenver.edu/DSigDB) to predict potential therapeutic drugs for IPAH, FDR < 0.05 and combined score > 5000 was used as the cutoff.

## 3. Results

### 3.1. Overall Protocol of the Study

**Figure 1:**
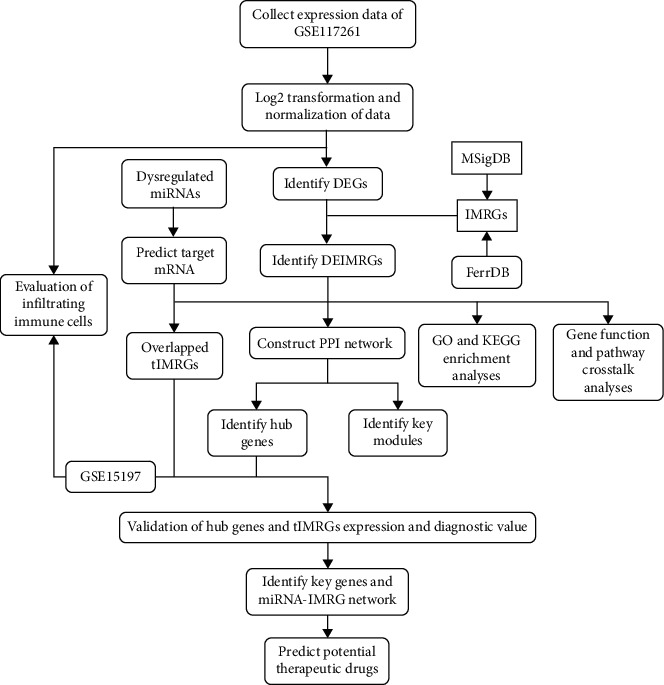
The overall protocol of this study. DEG: differentially expressed genes; IMRG: iron metabolism-related gene; DEIMRG: differentially expressed IMRG; tIMRG: target differentially expressed IMRG; DRmiRNA: differentially expressed microRNA; PPI: protein-protein interaction.

The overall flowchart of the study is summarized in Figure 1. All the raw data were log-transformed and quantized before analysis, as shown in Figure S1.

### 3.2. Identification of DEGs and DEIMRGs

**Figure 2:**
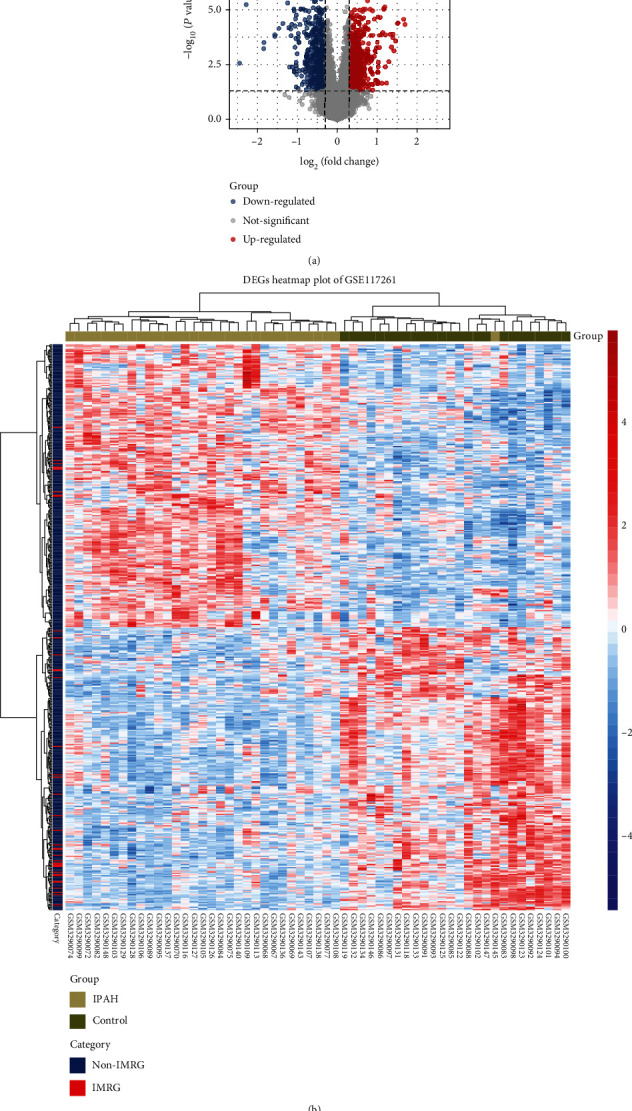
Identification of differentially expressed genes in GSE117261: (a) volcano plot of DEGs in GSE117261 (FDR < 0.05 and ∣log2FC | ≥0.25); (b) clustered heat map of DEGs in GSE117261 (FDR < 0.05 and ∣log2FC | ≥0.25). DEG: differentially expressed genes; IMRG: iron metabolism-related gene; Non-IMRG: noniron metabolism-related gene.

A total of 1526 DEGs were identified from GSE117261, of which 777 were upregulated and 749 were downregulated (Figure 2(a)). The clustered heat map of DEGs revealed that gene expression between the IPAH and control lung samples was distinct (Figure 2(b)).

**Figure 3:**
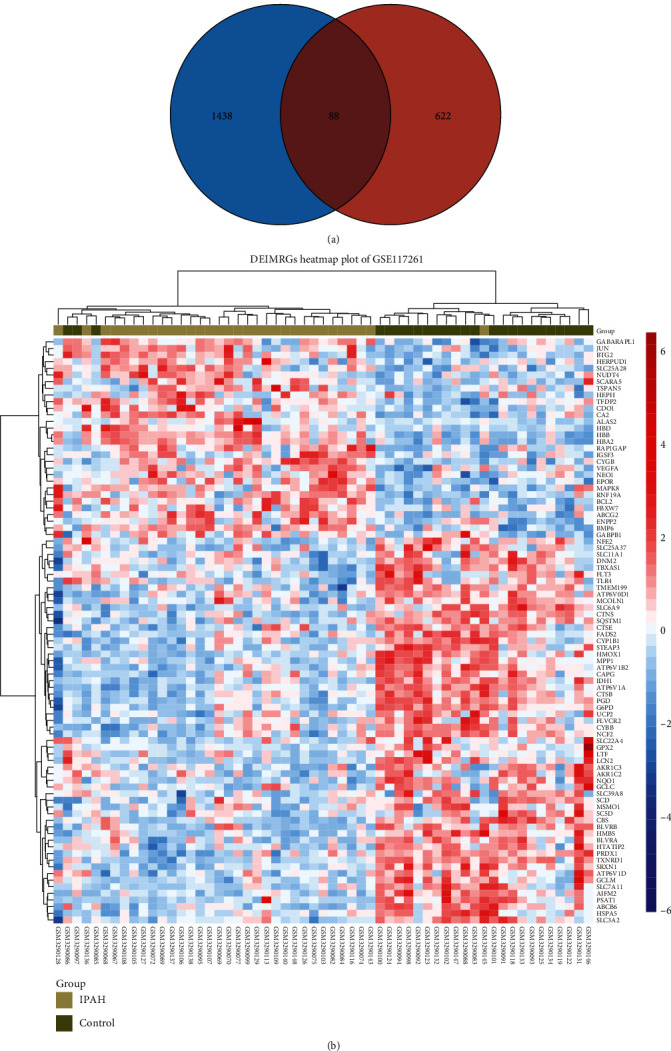
Identification of differentially expressed iron metabolism-related genes: (a) Venn diagram showing the overlap of genes between DEGs and IMRGs; (b) clustered heat map of DEIMRGs. DEG: differentially expressed genes; IMRG: iron metabolism-related gene; DEIMRG: differentially expressed IMRG.

After deduplication of genes, a total of 710 IMRGs were identified from MSigDb and FerrDb. We overlapped IMRGs with the DEGs in GSE117261 and selected 88 overlapped DEIMRGs for further analyses (Figure 3(a)), as listed in Table S3. The clustered heat map and correlation heat map were based on Ward.D2 algorithm showing the expression differences of 88 DEIMRGs between IPAH and control lung samples, as well as the correlation between DEIMRGs (Figure 3(b), S2).

### 3.3. GO and KEGG Enrichment Analyses

**Figure 4:**
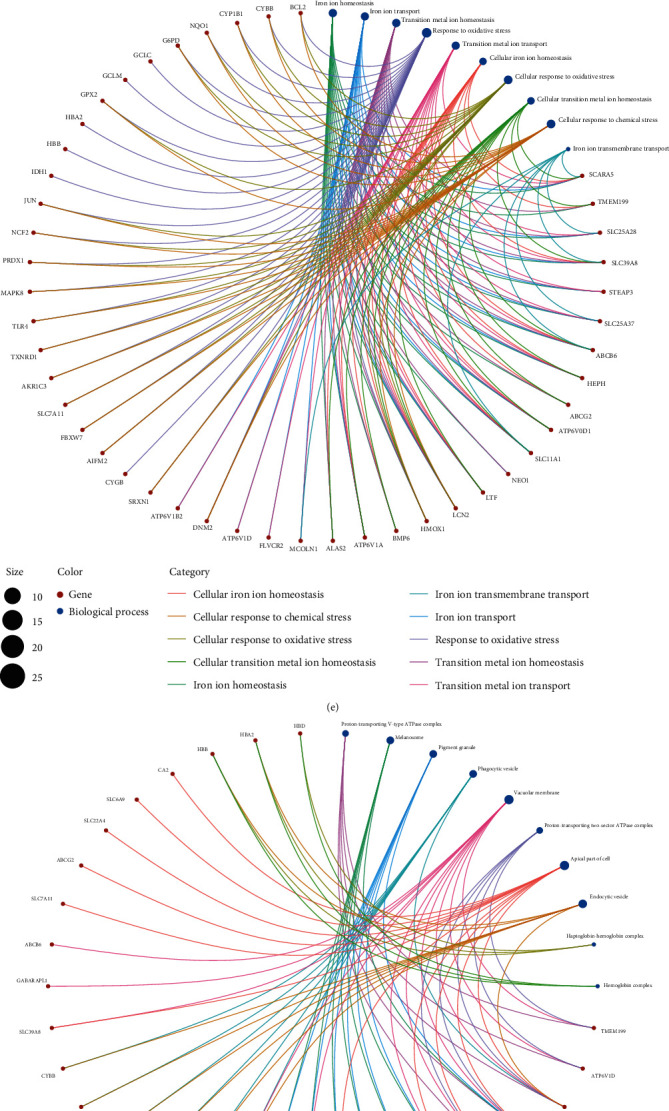
GO and KEGG enrichment analyses of DEIMRGs. GO enrichment analysis of DEIMRGs in (a) the biological process category (BP); (b) the cellular component category (CC); (c) the molecular function category (MF). (d) KEGG enrichment analysis of DEIMRGs. Crosstalk analysis between DEIMRGs and (e) gene functions in BP; (f) gene functions in CC; (g) gene functions in MF; (h) KEGG pathways. IMRG: iron metabolism-related gene; DEIMRG: differentially expressed IMRG.

We conducted GO and KEGG enrichment analyses to understand the functions and related pathways of the DEIMRGs. In the GO enrichment analysis, DEIMRGs were mainly enriched in response to oxidative stress and iron ion homeostasis in the biological process category (BP) (Figure 4(a)); vacuolar membrane and apical part of cell in the cellular component category (CC) (Figure 4(b)); and active transmembrane transporter activity and heme binding in the molecular function category (MF) (Figure 4(c)).In the KEGG enrichment analysis, most of the DEIMRPs participated in ferroptosis and fluid shear stress and atherosclerosis pathway (Figure 4(d)). We further analyzed the crosstalk between gene functions and pathways; the results suggesting that the role of IMRGs in the regulation of IPAH may be the result of the crosstalk of multiple gene functions and pathways, as shown in Figures 4(e)–4(h).

### 3.4. Prediction of Target Genes and Construction of DRmiRNA-DEIMRG Regulatory Network

**Figure 5:**
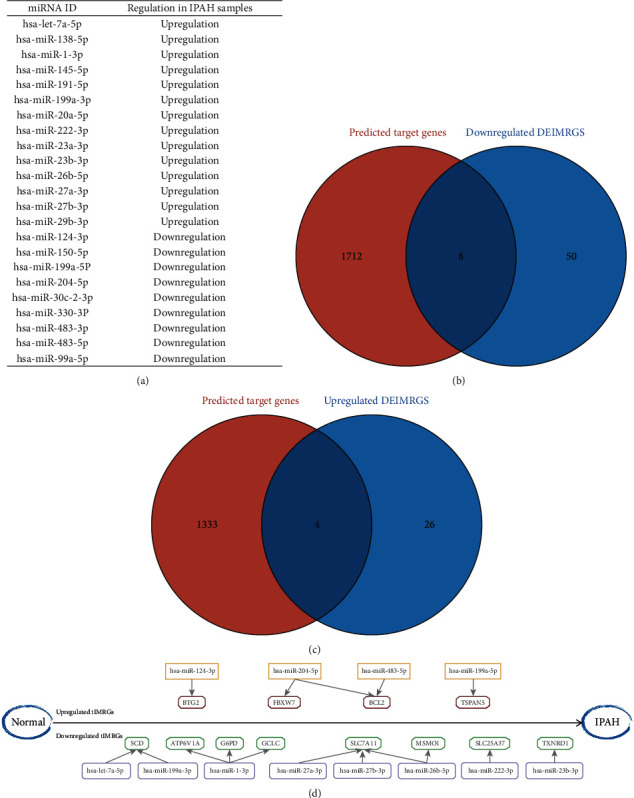
Prediction of target genes and construction of DRmiRNA-DEIMRG regulatory network: (a) list of DRmiRNAs; (b) Venn diagram showing the overlap of genes between upregulated DRmiRNAs and downregulated DEIMRGs; (c) Venn diagram showing the overlap of genes between downregulated DRmiRNAs and upregulated DEIMRGs; (d) DRmiRNA-tIMRG regulatory network in IPAH. DRmiRNAs: differentially expressed microRNAs; tIMRG: target differentially expressed IMRG.

After obtaining the DRmiRNAs as mentioned above, the corresponding target genes were predicted using the mirDIP database; the miRNA-mRNA regulatory network was constructed and presented (Figures S3 and S4); all DRmiRNAs are shown in Figure 5(a). We overlapped the 1337 and 1720 target genes predicted by 14 upregulated DRmiRNAs and 9 downregulated DRmiRNAs with 58 downregulated and 30 upregulated DEIMRGs, respectively (Figures 5(b) and 5(c)). The results showed that 8 downregulated DEIMRGs (SCD, ATP6V1A, G6PD, GCLC, SLC7A11, MSMO1, SLC25A37, and TXNRD1) were negatively regulated by 8 upregulated DRmiRNAs (let-7a-5p, miR-199a-3p, miR-1-3p, miR-27a-3p, miR-27b-3p, miR-26b-5p, miR-222-3p, and miR-23b-3p), and 4 upregulated DEIMRGs (BTG2, FBXW7, BCL2, and TSPAN5) were the target genes of 4 downregulated DRmiRNAs (miR-124-3p, miR-204-5p, miR-483-5p, and miR-199a-5p), as shown in Figure 5(d).

### 3.5. Construction of PPI Network and Identification of Key Modules and Hub Genes

**Figure 6:**
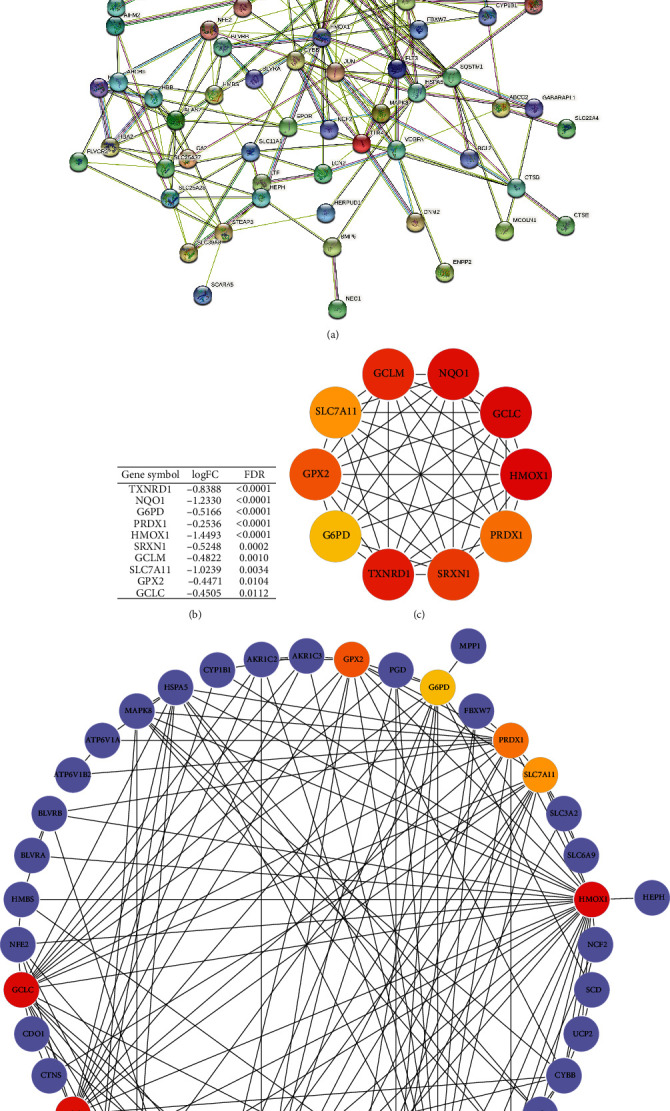
PPI network construction and identification of key modules and hub genes: (a) PPI network of DEIMRGs constructed by the STRING database; (b) the differential expression of hub genes in IPAH lung samples; (c) crosstalk between 10 hub genes; the deeper colour of the dot means that the rank order of the hub gene is more advanced; (d) crosstalk between 10 hub genes and other DEIMRGs. DEIMRG: differentially expressed IMRG.

To explore the interactions of these identified DEIMRGs, we constructed a PPI network (Figure 6(a)) of DEIMRGs using the STRING database. Further, we used Cytoscape software to analyze the data and identify key modules and hub genes. Finally, 3 key modules (Figure S5) were identified, and TXNRD1, NQO1, G6PD, PRDX1, HMOX1, SRXN1, GCLM, SLC7A11, GPX2, and GCLC were selected as hub genes; the rank values of all DEIMRGs are listed in Table S4. The differential expression of hub genes in IPAH lung samples is shown in Figure 6(b), while the multiple associations between hub genes and with other DEIMRGs are shown in Figures 6(c) and 6(d). Interestingly, module 1 overlaps exactly with the hub genes we identified, which further demonstrates that these hub genes are the major functional clusters in DEIMRGs.

### 3.6. Validation of the Expression and Diagnostic Value of Hub Genes and tIMRGs in GSE15197

**Figure 7:**
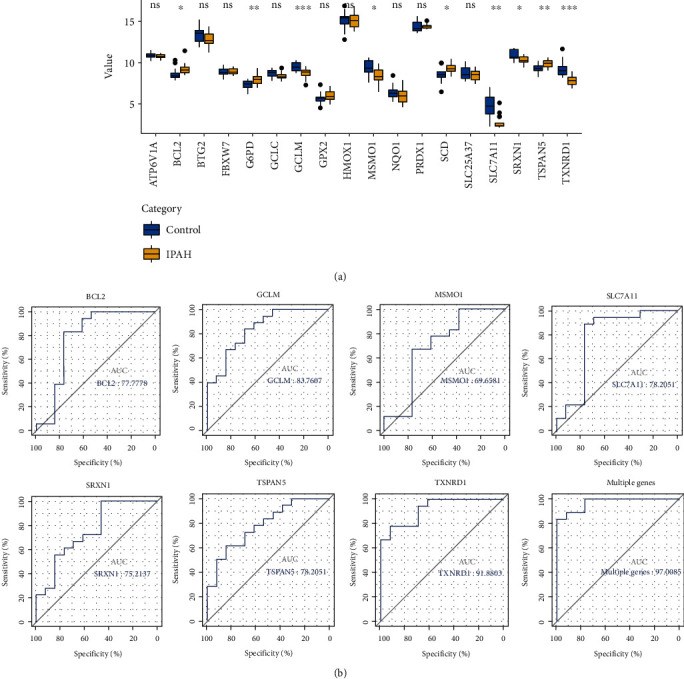
Validation of hub genes and target DEIMRG expression and diagnostic value in GSE15197. (a) Expression levels of hub genes and tIMRGs in IPAH and normal lung samples in GSE15197. (b) Receiver operating characteristic (ROC) analysis showed the predictive performance of hub genes for IPAH in GSE15197. AUC: area under the ROC curve; DEIMRG: differentially expressed IMRG. ^∗^*p* < 0.05, ^∗∗^*p* < 0.01, ^∗∗∗^*p* < 0.001, and ^∗∗∗∗^*p* < 0.0001; ns: not significant.

After normalization of the data from GSE15197, the expression data of 10 selected hub genes and 12 tIMRGs were extracted and statistically analyzed. Seven genes (BCL2, GCLM, MSMO1, SLC7A11, SRXN1, TSPAN5, and TXNRD1) showed the same trend of differential expression in IPAH samples as in GSE117261 (Figure 7(a)). Analysis of ROC curves showed that these 7 genes are of significant value for the diagnosis of IPAH, and the ROC curves of the multigene combinations including these 7 genes showed excellent predictive capability for IPAH (AUC = 97%), as shown in Figure 7(b). Therefore, we identified BCL2, GCLM, MSMO1, SLC7A11, SRXN1, TSPAN5, and TXNRD1 as key genes and constructed a miRNA-tIMRG regulatory network consisting of 6 miRNAs (miR-483-5p, miR-27a-3p, miR-27b-3p, miR-26b-5p, miR-199a-5p, and miR-23b-3p) with 5 IMRGs (BCL2, MSMO1, SLC7A11, TSPAN5, and TXNRD1).

### 3.7. Immune Infiltration Analyses

**Figure 8:**
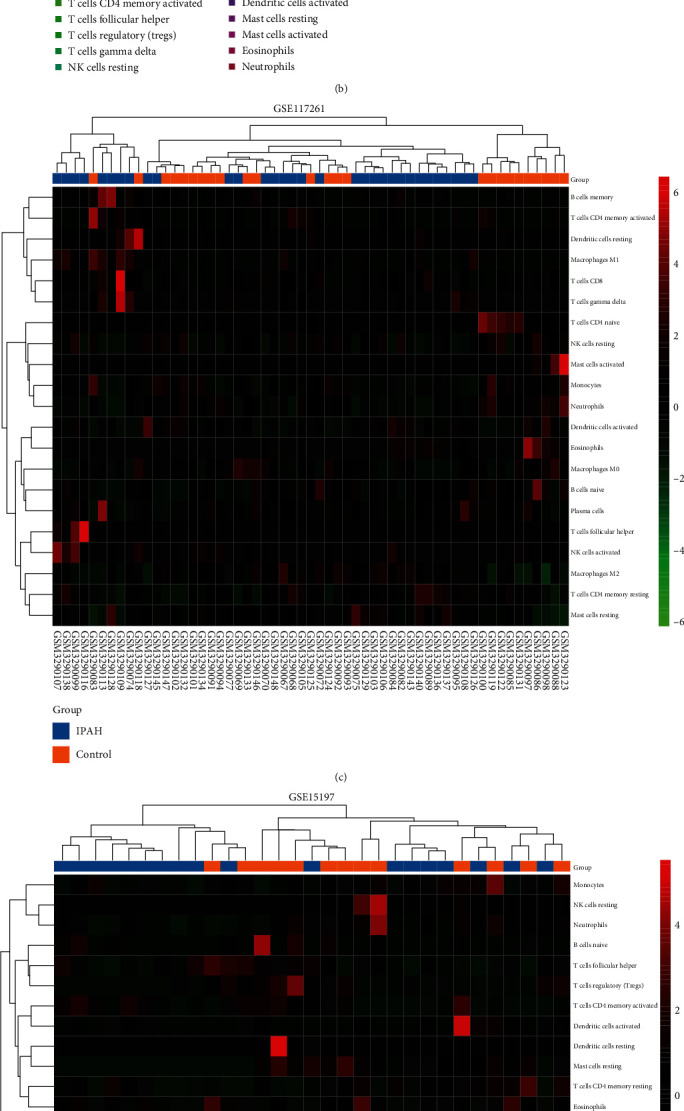
Immune infiltration analyses: (a) the proportion of infiltrating immune cells of the samples from GSE117261 by the CIBERSORT algorithm; (b) the proportion of infiltrating immune cells of the samples from GSE15197 by the CIBERSORT algorithm; (c) the clustering heat map of infiltrating immune cells in GSE117261; (d) the clustering heat map of infiltrating immune cells in GSE15197; (e) comparison of infiltrating immune cells between normal and IPAH lung tissues in GSE117261; (f) comparison of infiltrating immune cells between normal and IPAH lung tissues in GSE15197. ^∗^*p* < 0.05, ^∗∗^*p* < 0.01, ^∗∗∗^*p* < 0.001, and ^∗∗∗∗^*p* < 0.0001; ns: not significant.

We performed an immune infiltration analysis in an attempt to explore the crosstalk between iron metabolism and immune responses in IPAH. The proportion of infiltrating immune cells of the samples from the GSE117261 and GSE15197 datasets was estimated by the CIBERSORT algorithm (Tables S5 and S6) and then visualized (Figures 8(a) and 8(b)). The clustering heat map showed the difference between IPAH and control lung samples of infiltrating immune cells in the two datasets (Figures 8(c) and 8(d)), and correlation heat maps showed correlations between different infiltrating immune cells (Figure S6). In both datasets, the proportion of CD8^+^ T cells increased significantly in the IPAH samples and the proportion of neutrophils decreased significantly in the IPAH samples, while the other immune cells did not exhibit significant differences with a consistent trend (Figures 8(e) and 8(f)). The results of linear regression analysis showed that the expression of all key genes in both datasets did not show a significant correlation with the proportion of immune cells in the control samples (*p* > 0.05). As for IPAH samples, in the GSE117261 dataset, the expression of MSMO1 showed a significant positive correlation with the proportion of neutrophils, and the expression of TSPAN5 showed a significant positive correlation with the proportion of CD8^+^ T cells; in the GSE15197 dataset, the expression of GCLM, MSMO1, and TXNRD1 showed a significant negative correlation with the proportion of CD8^+^ T cells. Interestingly, none of the key genes we identified showed significant correlation with CD8^+^ T cells or neutrophils in both datasets. (Figure S7).

### 3.8. Targeted Drug Prediction

We used the DSigDB database to predict potential target drugs which are related to key genes, which may potentially treat IPAH by modulating iron metabolism. Finally, 34 target drugs were predicted; combined score and corresponding target genes are listed in Table S7. Figure S8 shows the top 10 predicted target drugs ranked according to FDR; the top two drugs—celastrol (combined score = 12028) and cinnamaldehyde (combined score = 6513) have a strong drug-target correlation (FDR < 0.0001).

## 4. Discussion

For IPAH, as a poor prognosis type of PAH, none of the current therapies are actually curative [4, 6, 7]. However, targeted therapies for specific genes, such as BMPR2, in patients with IPAH have shown some encouraging results [24, 25], indicating that further exploration of the molecular and pathological mechanisms of IPAH may provide promising therapeutic targets for patients. Dysregulated iron metabolism is closely associated with the development and progression of various cardiovascular diseases, including coronary artery disease, heart failure, and pulmonary hypertension [26]. A large proportion of patients with IPAH are characterized by iron deficiency, even without anemia, and associated with reduced exercise capacity and survival [10–12], suggesting that dysregulation of iron homeostasis may be a potential mechanism for the development and progression of IPAH. However, whether iron deficiency contributes to or is merely a consequence of IPAH remains debated; the mechanisms by which dysregulated iron metabolism participates in the development of IPAH are still unclear. Thus, we performed a bioinformatic analysis based on IPAH-related datasets to explore the role of iron metabolism on the development of IPAH.

The results of differential expression analysis showed that a significant proportion of IMRGs were differentially expressed in IPAH and normal lung samples. Further, GO enrichment analysis revealed that the molecular function of IMRGs is mainly enriched in active transmembrane transporter activity and mainly affects the biological process of response to oxidative stress. Several studies have indicated that there is an abnormal elevation of hepcidin in IPAH patients due to various factors such as BMPR2 mutation and inflammatory response, which can inhibit intestinal iron uptake and intracellular iron export, leading to circulating iron deficiency and intracellular iron overload [12, 27–29]. Intracellular iron overload is associated with mitochondrial dysfunction and production of reactive oxygen species and causes lipid peroxidation, DNA oxidation, and protein oxidation such as carbonylation, via the Fenton reaction and the Haber-Weiss pathway, and hence affects the cellular response to oxidative stress [27, 30–32], which has been demonstrated to be an important biological process involved in the progression of IPAH by affecting pulmonary vascular function and remodeling [33–36]. KEGG enrichment analysis identified pathways that may be involved in the regulation of DEIMRGs—ferroptosis and fluid shear stress and atherosclerosis pathway. Theoretically, the activation of ferroptosis pathway may be associated with iron-dependent lipid peroxidation induced by intracellular iron overload and lead to pulmonary vascular remodeling by affecting protein carbonylation [30, 37, 38], while fluid shear stress may cause vascular remodeling through iron-mediated generation of atherogenic mediators [39]. However, the role of these pathways involved in mediating iron metabolism dysregulation on the pathogenesis of IPAH needs to be further explored, as there are few relevant studies.

miRNAs play an important regulatory role in the development of IPAH and have been demonstrated to be involved in the progression of IPAH by regulating the expression of target genes affecting metabolism and proliferation, DNA damage, vasoconstriction, and angiogenesis [40–42]. Iron metabolism has been reported to be regulated by miRNAs, which have been demonstrated to posttranscriptionally regulate the expression of genes associated with iron acquisition, iron export, iron storage, iron utilization, and coordination of systemic iron homeostasis [43–45]. We found that some dysregulated miRNAs in IPAH patients regulate iron metabolism in other biological circumstances [44, 46–48]; given that both iron metabolism disorders and miRNA dysregulation are important regulators of IPAH development, we hypothesized that there is crosstalk between them in IPAH patients and then constructed a DRmiRNA-DEIMRG regulatory network and identified 12 tIMRGs. In addition, we identified 10 hub genes in DEIMRGs that are highly associated with other proteins through the construction of PPI networks. After validation of tIMRGs and hub genes in another independent dataset, we identified 7 key genes associated with iron metabolism. Intracellular iron overload leads to reduced expression of GCLM and SLC7A11, which consequently affects glutathione synthesis or intracellular unstable iron metabolism, resulting in cellular ferroptosis [49, 50]. The NRF2 signaling pathway plays a critical role in mitigating lipid peroxidation and ferroptosis, whereas the downregulation of TXNRD1 and SRXN1, important signaling molecules in the NRF2 signaling pathway, may render cells more susceptible to ferroptosis. Although studies have reported increased expression of TXNRD1 and SRXN1 in protective iron overload heart and kidney tissues due to activation of the NRF2 signaling pathway, their expression was decreased in IPAH lung samples we analyzed, which may be due to much higher levels of iron overload and the presence of oxidative stress activated by other factors [51–55]. The increased expression of TSPAN5 may be related to the activation of the NOTCH signaling pathway by increased cellular uptake of iron, while the downregulation of MSMO1 expression may be mechanistically related to heme metabolism, but relevant studies are lacking [56–59]. In addition, intracellular iron overload usually leads to downregulation of BCL2 and induces apoptosis; interestingly, the expression of BCL2 was upregulated in the IPAH lung samples we analyzed, which may result in abnormal antiapoptotic phenotypic changes in pulmonary vascular endothelial cells and pulmonary vascular smooth muscle cells due to factors other than iron metabolism [60–62]. Five of the key genes (BCL2, MSMO1, SLC7A11, TSPAN5, and TXNRD1) as target genes may be regulated by 6 DRmiRNAs (miR-483-5p, miR-27a-3p, miR-27b-3p, miR-26b-5p, miR-199a-5p, and miR-23b-3p), which could be a potential crosstalk between iron metabolism and miRNA regulation in IPAH. Several key genes have been reported to be involved in the development of IPAH, but their iron metabolism-related regulatory mechanisms in IPAH patients remain unclear, as well as their regulation by miRNAs, which needs to be further explored [57].

It is well known that immune and inflammatory responses play a crucial role in the pathogenesis of IPAH [63, 64], while genetic and metabolic abnormalities are inextricably linked to dysregulated immunity and adverse remodeling in the pulmonary arteries [65]. Recent studies have shown that iron homeostasis plays an important role in the regulation of immune responses, and imbalance of iron homeostasis may affect the development, function, and death of immune cells [66]. The immune infiltration analysis in our study showed a significantly increased proportion of CD8^+^ T cells and a significantly decreased proportion of neutrophils in IPAH lung samples, which is consistent with previous reports [64, 67, 68]. Although there was a linear correlation between some key genes and CD8^+^ T cells and neutrophils, this correlation did not show consistency in both datasets, which may be due to the limitation or individual differences of the regulation of immune infiltration in IPAH by IMRGs, and the regulation of iron metabolism in IPAH on immune infiltration requires further research.

The exploration of effective target therapeutics based on genes that play a key role in pathology has always been the focus of researchers [25, 69]. According to the key genes we identified, we predicted several potential targeting drugs, especially celastrol and cinnamaldehyde, which showed high drug-targeting correlations. Cinnamaldehyde treatment can inhibit MCT-induced elevation in right ventricle systolic pressure, RV/LV + S, and right ventricular collagen accumulation. Celastrol treatment can ameliorate right ventricular systolic pressure, hypertrophy, fibrosis, and dysfunction in hypoxia-induced PAH in mice and SU5416/hypoxia-induced PAH in rats. Although both drugs were identified to be protective against PAH, modulation of iron metabolism as its potential functional mechanism has not been explored; further experimental clarification is needed.

To define the role of dysregulated iron metabolism in IPAH, further validation of our results in an appropriate animal model is necessary but difficult. Most current animal models of PAH have been constructed by chemical induction, chronic hypoxia, or surgery. Due to the considerable pathological differences between different species of PAH, although these animal models morphologically reproduce the features of human PAH, there are currently no available animal models that well reproduce the histological features and natural history of IPAH, which makes the validation potentially inaccurate and even contradictory conclusions. Morphological research and validation of protein expression levels on large human samples are urgently needed for further studies.

## 5. Conclusion

We identified DEIMRGs in normal and IPAH lung samples and analyzed their potential regulatory mechanisms and further identified key genes. In addition, we found that IMRGs may be regulated by miRNAs and then identified crucial miRNA-IMRG regulatory networks. These findings contribute to a deeper understanding of the unique role of dysregulated iron metabolism in IPAH, and in-depth studies of IMRG may provide potential therapeutic targets and biomarkers for IPAH patients, yet further studies are needed to analyze the complex regulatory mechanisms.

## Data Availability

The microarray datasets GSE113439 and GSE117261 were downloaded from the Gene Expression Omnibus (GEO) database (https://www.ncbi.nlm.nih.gov/geo/). IMRGs were identified from related gene sets (GOBP_IRON_ION_HOMEOSTASIS, GOBP_IRON_ION_TRANSPORT, GOBP_HEME_METABOLIC_PROCESS, GOBP_RESPONSE_TO_IRON_ION, GOBP_IRON_SULFUR_CLUSTER_ASSEMBLY, HALLMARK_HEME_METABOLISM, GOMF_IRON_ION_BINDING, REACTOME_IRON_UPTAKE_AND_TRANSPORT, and HP_ABNORMALITY_OF_IRON_HOMEOSTASIS) in the MSigDb database (https://www.gsea-msigdb.org/gsea/msigdb/index.jsp) and ferroptosis-related genes in the FerrDb database (http://www.zhounan.org/ferrdb). The mirDIP database (http://ophid.utoronto.ca/mirDIP/) was used to predict the target genes of DE-miRNAs. The STRING database (https://string-db.org/) was used to construct a PPI network. The DSigDB database was used to predict potential therapeutic drugs, and the drug structures were obtained from the DrugBank database (https://www.drugbank.ca/).

## Supplementary Materials

Supplementary Materials Supplementary materials are available online at DOI: 10.6084/m9.figshare.14877513. Figure S1: gene expression vioplot of GSE117261 and GSE15197 after normalization. Figure S2: correlation heat map of differentially expressed iron metabolism-related genes in GSE117261. Figure S3: predicted target genes of downregulated miRNA. Figure S4: predicted target genes of upregulated miRNA. Figure S5: key modules identified by the Cytoscape plugin MCODE. Table S1: the merged iron metabolism-related gene set. Figure S6: correlation heat map of immune cells in GSE117261 and GSE15197. Figure S7: linear regression analysis between expression of key genes and the proportion of immune cells in GSE117261 and GSE15197. Figure S8: top 10 targeted drugs predicted in the DSigDB database ranked by FDR. Table S1: the merged iron metabolism related gene set. Table S2: dysregulated miRNAs in IPAH samples. Table S3: differentially expressed iron metabolism-related gene set. Table S4: rank values of differentially expressed iron metabolism-related genes by MCC algorithm. Table S5: the proportion of infiltrating immune cells estimated by the CIBERSORT algorithm in GSE117261. Table S6: the proportion of infiltrating immune cells estimated by the CIBERSORT algorithm in GSE15197. Table S7: predicted target drug using the DSigDB database.
