# Supplementary material for: Iron Metabolism and Idiopathic Pulmonary Arterial Hypertension: New Insights from Bioinformatic Analysis
Source: Biomed Res Int. 2021 Oct 22;2021:5669412. doi: 10.1155/2021/5669412 (PMC8556088; doi:10.1155/2021/5669412)
Supplement: Supplementary Materials — are available online at DOI: 10.6084/m9.figshare.14877513. Figure S1: gene expression vioplot of GSE117261 and GSE15197 after normalization. Figure S2: correlation heat map of differentially expressed iron metabolism-related genes in GSE117261. Figure S3: predicted target genes of downregulated miRNA. Figure S4: predicted target genes of upregulated miRNA. Figure S5: key modules identified by the Cytoscape plugin MCODE. Table S1: the merged iron metabolism-related gene set. Figure S6: correlation heat map of immune cells in GSE117261 and GSE15197. Figure S7: linear regression analysis between expression of key genes and the proportion of immune cells in GSE117261 and GSE15197. Figure S8: top 10 targeted drugs predicted in the DSigDB database ranked by FDR. Table S1: the merged iron metabolism related gene set. Table S2: dysregulated miRNAs in IPAH samples. Table S3: differentially expressed iron metabolism-related gene set. Table S4: rank values of differentially expressed iron metabolism-related genes by MCC algorithm. Table S5: the proportion of infiltrating immune cells estimated by the CIBERSORT algorithm in GSE117261. Table S6: the proportion of infiltrating immune cells estimated by the CIBERSORT algorithm in GSE15197. Table S7: predicted target drug using the DSigDB database. [file 5669412.f1.zip › Table S1 The merged iron metabolism related gene set (1).pdf]

Table S1 The merged iron metabolism related gene set

Gene symbol
YPEL5
XPO7
XK
XDH
VEZF1
USP15
UROS
UROD
UGT1A4
UGT1A1
UCP2
UBC
UBB
UBAC1
UBA52
TYW5
TYR
TTYH1
TTC7A
TTC37
TSPO2
TSPO
TSPAN5
TRNT1
TRIM58
TRIM10
TRAK2
TPH2
TPH1
TOP1
TNS1
TNRC6B
TMPRSS6
TMLHE
TMEM9B
TMEM199
TMEM14C
TMCC2
TH
TFRC
TFR2
TFF1
TFDP2
TFAP2A

TF
TET2
TET1
TENT5C
TCIRG1
TCEA1
TBXAS1
TAL1
SYNJ1
STEAP4
STEAP3
STEAP2
STEAP1
SRRD
SRI
SPTB
SPTA1
SOD1
SNCA
SMOX
SMAD4
SLC7A11
SLC6A9
SLC6A8
SLC6A3
SLC66A2
SLC4A1
SLC48A1
SLC46A1
SLC40A1
SLC39A8
SLC39A14
SLC30A10
SLC30A1
SLC2A1
SLC25A39
SLC25A38
SLC25A37
SLC25A28
SLC22A4
SLC22A17
SLC11A2
SLC11A1
SLC10A3
SKP1
SKIV2L
SIDT2

SELENBP1
SEC14L1
SDCBP
SCD5
SCD
SCARA5
SC5D
RPS27A
RNF19A
RNF123
RIOX1
RIOK3
RHD
RHCE
RHAG
REP15
RCL1
RBM5
RBM38
RAP1GAP
RANBP10
RAD23A
RAB11B
PTGIS
PSMD9
PRDX2
PPP2R5B
PPOX
PPEF2
PPEF1
PLOD3
PLOD2
PLOD1
PKLR
PIGQ
PICALM
PHYH
PHF8
PHF2
PGRMC2
PGRMC1
PGLS
PDZK1IP1
PDX1
PC
PAH
P4HTM

P4HA3
P4HA2
P4HA1
P3H3
P3H2
P3H1
OSBP2
OPTN
OGFOD3
OGFOD2
OGFOD1
NUDT4
NUBPL
NUBP2
NUBP1
NR3C1
NOX5
NNT
NFU1
NFS1
NFE2L1
NFE2
NEO1
NEK7
NEDD8
NECTIN1
NDOR1
NDFIP1
NCOA4
NARF
MYL4
MYC
MXI1
MSMO1
MPP1
MOSPD1
MOCOS
MMS19
MMGT1
MKRN1
MIR210
MIOX
MINPP1
MGST3
MFHAS1
MELTF
MCOLN1

MBOAT2
MARK3
MARCHF8
MARCHF2
MAP2K3
MAP1LC3A
LYRM4
LTF
LRP10
LPIN2
LMTK2
LMO2
LCN2
LAMP2
KLF3
KLF1
KIF23
KHNYN
KEL
KDM7A
KDM3A
KAT2B
JMJD6
ISCU
ISCA2
ISCA1
IREB2
IGSF3
IFNG
ICAM4
IBA57
HYAL2
HTRA2
HTATIP2
HSPA9
HSCB
HRG
HPX
HMOX2
HMOX1
HMBS
HJV
HIF1AN
HIF1A
HFE
HEPHL1
HEPH

HEBP1
HDGF
HBZ
HBQ1
HBD
HBBP1
HBB
HBA2
HBA1
HAMP
HAGH
HAAO
H4C3
H1-0
GYPE
GYPC
GYPB
GYPA
GMPS
GLRX5
GLRX3
GDF2
GDE1
GCLM
GCLC
GATA1
GAPVD1
G6PD
FXN
FTO
FTMT
FTL
FTHL17
FTH1P19
FTH1
FTCD
FOXP1
FOXO3
FOXJ2
FN3K
FLVCR2
FLVCR1
FECH
FDX1
FBXO9
FBXO7
FBXO34

FBXL5
FAXDC2
FA2H
EZH1
ETHE1
ERMAP
ERFE
EPOR
EPB42
EPB41
EPAS1
ENDOD1
ELL2
EIF2AK1
EGLN3
EGLN2
EGLN1
E2F2
DRD2
DOHH
DNM2
DNAJC24
DMTN
DCUN1D1
DCAF11
DCAF10
DAAM1
CYP8B1
CYP7B1
CYP7A1
CYP51A1
CYP4Z2P
CYP4Z1
CYP4X1
CYP4V2
CYP4F8
CYP4F3
CYP4F22
CYP4F2
CYP4F12
CYP4F11
CYP4B1
CYP4A22
CYP4A11
CYP46A1
CYP3A7
CYP3A5

CYP3A43
CYP3A4
CYP39A1
CYP2W1
CYP2U1
CYP2S1
CYP2R1
CYP2J2
CYP2G1P
CYP2F1
CYP2E1
CYP2D7
CYP2D6
CYP2C9
CYP2C8
CYP2C19
CYP2C18
CYP2B6
CYP2A7
CYP2A6
CYP2A13
CYP27C1
CYP27B1
CYP27A1
CYP26C1
CYP26B1
CYP26A1
CYP24A1
CYP21A2
CYP20A1
CYP1B1
CYP1A2
CYP1A1
CYP19A1
CYP17A1
CYP11B2
CYP11B1
CYP11A1
CYGB
CYBRD1
CUL1
CTSE
CTSB
CTNS
CROCCP2
CPOX
CP

COX15
COX10
COL7A1
CLTC
CLIC2
CLCN3
CIR1
CIAPIN1
CIAO3
CIAO2B
CIAO2A
CIAO1
CH25H
CDR2
CDO1
CDC27
CCND3
CCND1
CCNB1
CCDC28A
CCDC115
CAT
CAST
CARD9
CAND1
CALR
CA2
CA1
C3
C1QA
C1orf194
BTRC
BTG2
BTBD9
BSG
BPGM
BOLA2B
BOLA2
BNIP3L
BMP6
BMP2K
BMP2
BLVRB
BLVRA
BECN1
BDH2
BCS1L

BCL2
BCAM
BBOX1
BACH1
B2M
ATP7A
ATP6V1H
ATP6V1G3
ATP6V1G2
ATP6V1G1
ATP6V1F
ATP6V1E2
ATP6V1E1
ATP6V1D
ATP6V1C2
ATP6V1C1
ATP6V1B2
ATP6V1B1
ATP6V1A
ATP6V0E2
ATP6V0E1
ATP6V0D2
ATP6V0D1
ATP6V0C
ATP6V0B
ATP6V0A4
ATP6V0A2
ATP6V0A1
ATP6AP1
ATP5IF1
ATP13A2
ATG4A
ASNS
ASIC3
ARL2BP
ARHGEF12
ARHGAP1
AQP3
AOX1
ANK1
AMBP
ALOXE3
ALOX5
ALOX15B
ALOX15
ALOX12B
ALOX12

ALKBH8
ALKBH3
ALKBH2
ALKBH1
ALDH6A1
ALDH1L1
ALAS2
ALAS1
ALAD
AHSP
AGPAT4
AGMO
ADIPOR1
ADI1
ADD2
ADD1
ACSL6
ACP5
ACO2
ACO1
ACKR1
ABCG2
ABCE1
ABCB7
ABCB6
RPL8
ATP5MC3
CS
EMC2
ACSF2
NOX1
CYBB
NOX3
NOX4
DUOX1
DUOX2
PGD
VDAC2
PIK3CA
FLT3
SCP2
TP53
ACSL4
LPCAT3
NRAS
KRAS
HRAS

SLC38A1
SLC1A5
GLS2
GOT1
CARS1
KEAP1
ATG5
ATG7
PHKG2
G6PDX
ULK1
ATG3
ATG4D
GABARAPL2
GABARAPL1
ATG16L1
WIPI1
WIPI2
SNX4
ATG13
ULK2
SAT1
EGFR
MAPK3
MAPK1
BID
ZEB1
DPP4
CDKN2A
PEBP1
SOCS1
MYB
MAPK8
MAPK9
CHAC1
MAPK14
LINC00472
PRKAA2
PRKAA1
ELAVL1
BAP1
ABCC1
MIR6852
ACVR1B
TGFBR1
HILPDA
ANO6

LPIN1
HMGB1
TNFAIP3
TLR4
ATF3
ATM
YY1AP1
TAZ
MTDH
IDH1
SIRT1
FBXW7
PANX1
DNAJB6
LONP1
PTGS2
DUSP1
NOS2
NCF2
MT3
ALB
TXNRD1
SRXN1
GPX2
BNIP3
OXSR1
SELENOS
ANGPTL7
DDIT4
LOC284561
TSC22D3
DDIT3
JDP2
SESN2
SLC1A4
PCK2
TXNIP
VLDLR
GPT2
PSAT1
LURAP1L
SLC7A5
HERPUD1
XBP1
SLC3A2
CBS
ATF4

ZNF419
KLHL24
TRIB3
ZFP69B
VEGFA
GDF15
TUBE1
ARRDC3
CEBPG
SNORA16A
RGS4
BLOC1S5-TXNDC5
LOC390705
EIF2S1
KIM-1
IL6
CXCL2
RELA
HSD17B11
AGPAT3
SETD1B
MAFG
IL33
GPX4
HSPB1
NFE2L2
DRD5
DRD4
MAP3K5
SLC2A3
SLC2A6
SLC2A8
SLC2A12
GLUT13
SLC2A14
EIF2AK4
TFAP2C
SP1
NNMT
PLIN4
HIC1
STMN1
RRM2
CAPG
HNF4A
NGB
YWHAE

GABPB1
AURKA
MIR4715
RIPK1
PRDX1
MIR30B
AKR1C1
AKR1C2
AKR1C3
RB1
HSF1
SQSTM1
NQO1
MUC1
MT1G
CISD1
FANCD2
HSPA5
HELLS
FADS2
SRC
STAT3
PML
MTOR
TP63
CDKN1A
MIR137
ENPP2
FH
CISD2
MIR9-1
MIR9-2
MIR9-3
ACSL3
OTUB1
CD44
LINC00336
BRD4
PRDX6
MIR17
NF2
ARNTL
JUN
CA9
TMBIM4
PLIN2
MIR212

Fer1HCH
AIFM2
ZFP36
PROM2
CHMP5
CHMP6
CAV1
GCH1
