# Supplementary material for: Iron Metabolism and Idiopathic Pulmonary Arterial Hypertension: New Insights from Bioinformatic Analysis
Source: Biomed Res Int. 2021 Oct 22;2021:5669412. doi: 10.1155/2021/5669412 (PMC8556088; doi:10.1155/2021/5669412)
Supplement: Supplementary Materials — are available online at DOI: 10.6084/m9.figshare.14877513. Figure S1: gene expression vioplot of GSE117261 and GSE15197 after normalization. Figure S2: correlation heat map of differentially expressed iron metabolism-related genes in GSE117261. Figure S3: predicted target genes of downregulated miRNA. Figure S4: predicted target genes of upregulated miRNA. Figure S5: key modules identified by the Cytoscape plugin MCODE. Table S1: the merged iron metabolism-related gene set. Figure S6: correlation heat map of immune cells in GSE117261 and GSE15197. Figure S7: linear regression analysis between expression of key genes and the proportion of immune cells in GSE117261 and GSE15197. Figure S8: top 10 targeted drugs predicted in the DSigDB database ranked by FDR. Table S1: the merged iron metabolism related gene set. Table S2: dysregulated miRNAs in IPAH samples. Table S3: differentially expressed iron metabolism-related gene set. Table S4: rank values of differentially expressed iron metabolism-related genes by MCC algorithm. Table S5: the proportion of infiltrating immune cells estimated by the CIBERSORT algorithm in GSE117261. Table S6: the proportion of infiltrating immune cells estimated by the CIBERSORT algorithm in GSE15197. Table S7: predicted target drug using the DSigDB database. [file 5669412.f1.zip › Table S2 Dysregulated miRNAs in IPAH samples.pdf]

Table S2 Dysregulated miRNAs in IPAH samples

miRNA ID	Regulation in IPAH samples	Organism	Experiment type	Functional validation	PMID	Auther	Year
hsa-let-7a-5p	Up-regulation	Homo sapiens	miRNAs-microarray + qRT-PCR	No	27188753	Wu et al	2016
hsa-miR-124-3p	Down-regulation	Homo sapiens	miRNAs-microarray + qRT-PCR	Yes	28971999	Paola et al	2017
hsa-miR-124-3p	Down-regulation	Homo sapiens	qRT-PCR	Yes	24122720	Wang et al	2013
hsa-miR-138-5p	Up-regulation	Homo sapiens	miRNAs-microarray + qRT-PCR	Yes	31553627	Aleksandra et al	2019
hsa-miR-1-3p	Up-regulation	Homo sapiens	miRNAs-microarray + qRT-PCR	No	25815108	Irene et al	2015
hsa-miR-145-5p	Up-regulation	Homo sapiens	miRNAs-microarray + qRT-PCR	No	25815108	Irene et al	2015
hsa-miR-150-5p	Down-regulation	Homo sapiens	miRNAs-microarray + qRT-PCR	No	23220912	Christopher et al	2013
hsa-miR-150-5p	Down-regulation	Homo sapiens	qRT-PCR	Yes	33335799	Giusy et al	2020
hsa-miR-191-5p	Up-regulation	Homo sapiens	miRNAs-microarray + qRT-PCR	No	25815108	Irene et al	2015
hsa-miR-199a-3p	Up-regulation	Homo sapiens	miRNAs-microarray + qRT-PCR	No	27188753	Wu et al	2016
hsa-miR-199a-5P	Down-regulation	Homo sapiens	miRNAs-microarray + qRT-PCR	No	25815108	Irene et al	2015
hsa-miR-204-5p	Down-regulation	Homo sapiens	miRNAs-microarray + qRT-PCR	Yes	21321078	Audrey et al	2011
hsa-miR-204-5P	Down-regulation	Homo sapiens	miRNAs-microarray + qRT-PCR	No	25815108	Irene et al	2015
hsa-miR-20a-5p	Up-regulation	Homo sapiens	miRNAs-microarray + qRT-PCR	No	25815108	Irene et al	2015
hsa-miR-222-3p	Up-regulation	Homo sapiens	miRNAs-microarray + qRT-PCR	Yes	31553627	Aleksandra et al	2019
hsa-miR-223-3p	Up-regulation	Homo sapiens	miRNAs-microarray + qRT-PCR	Yes	30507047	Liu et al	2018
hsa-miR-23a-3p	Up-regulation	Homo sapiens	miRNAs-microarray + qRT-PCR	No	25815108	Irene et al	2015
hsa-miR-23b-3p	Up-regulation	Homo sapiens	miRNAs-microarray + qRT-PCR	No	25815108	Irene et al	2015
hsa-miR-26b-5p	Up-regulation	Homo sapiens	miRNAs-microarray + qRT-PCR	No	27188753	Wu et al	2016
hsa-miR-27a-3p	Up-regulation	Homo sapiens	miRNAs-microarray + qRT-PCR	No	25815108	Irene et al	2015
hsa-miR-27b-3p	Up-regulation	Homo sapiens	miRNAs-microarray + qRT-PCR	No	27188753	Wu et al	2016
hsa-miR-29b-3p	Up-regulation	Homo sapiens	miRNAs-microarray + qRT-PCR	Yes	31553627	Aleksandra et al	2019
hsa-miR-30c-2-3p	Down-regulation	Homo sapiens	miRNAs-microarray + qRT-PCR	No	25815108	Irene et al	2015
hsa-miR-330-3P	Down-regulation	Homo sapiens	miRNAs-microarray + qRT-PCR	No	25815108	Irene et al	2015
hsa-miR-483-3p	Down-regulation	Homo sapiens	RNA-sequencing + qRT-PCR	Yes	32324970	Zhang et al	2020
hsa-miR-483-5p	Down-regulation	Homo sapiens	RNA-sequencing + qRT-PCR	Yes	32324970	Zhang et al	2020
hsa-miR-99a-5p	Down-regulation	Homo sapiens	miRNAs-microarray + qRT-PCR	No	25815108	Irene et al	2015
