# Supplementary material for: Iron Metabolism and Idiopathic Pulmonary Arterial Hypertension: New Insights from Bioinformatic Analysis
Source: Biomed Res Int. 2021 Oct 22;2021:5669412. doi: 10.1155/2021/5669412 (PMC8556088; doi:10.1155/2021/5669412)
Supplement: Supplementary Materials — are available online at DOI: 10.6084/m9.figshare.14877513. Figure S1: gene expression vioplot of GSE117261 and GSE15197 after normalization. Figure S2: correlation heat map of differentially expressed iron metabolism-related genes in GSE117261. Figure S3: predicted target genes of downregulated miRNA. Figure S4: predicted target genes of upregulated miRNA. Figure S5: key modules identified by the Cytoscape plugin MCODE. Table S1: the merged iron metabolism-related gene set. Figure S6: correlation heat map of immune cells in GSE117261 and GSE15197. Figure S7: linear regression analysis between expression of key genes and the proportion of immune cells in GSE117261 and GSE15197. Figure S8: top 10 targeted drugs predicted in the DSigDB database ranked by FDR. Table S1: the merged iron metabolism related gene set. Table S2: dysregulated miRNAs in IPAH samples. Table S3: differentially expressed iron metabolism-related gene set. Table S4: rank values of differentially expressed iron metabolism-related genes by MCC algorithm. Table S5: the proportion of infiltrating immune cells estimated by the CIBERSORT algorithm in GSE117261. Table S6: the proportion of infiltrating immune cells estimated by the CIBERSORT algorithm in GSE15197. Table S7: predicted target drug using the DSigDB database. [file 5669412.f1.zip › Table S3 Differentially expressed iron metabolism related gene set.pdf]

Table S3 Differentially expressed iron metabolism related gene set

Gene symbol	logFC	FDR	Trend
HBB	2.573747149	2.71E-09	Up-regulated
HBA2	2.160057439	0.000000031	Up-regulated
ENPP2	1.361442229	0.000519091	Up-regulated
BMP6	1.167051595	0.00000944	Up-regulated
ABCG2	1.027422234	0.0000226	Up-regulated
CA2	0.793814377	3.60E-04	Up-regulated
CDO1	0.697527747	0.008809255	Up-regulated
BTG2	0.550681527	0.00833395	Up-regulated
JUN	0.519652719	0.013539847	Up-regulated
HBD	0.479259714	0.002693016	Up-regulated
VEGFA	0.406894794	0.004660859	Up-regulated
CYGB	0.361736735	0.000747666	Up-regulated
NEO1	0.355296307	0.017357097	Up-regulated
BCL2	0.342778806	0.00011371	Up-regulated
ALAS2	0.341265324	0.012706221	Up-regulated
HERPUD1	0.341166721	0.005484379	Up-regulated
TFDP2	0.332752471	8.03E-03	Up-regulated
SCARA5	0.332673388	0.012683785	Up-regulated
HEPH	0.322354625	0.038237251	Up-regulated
IGSF3	0.303331846	2.20E-02	Up-regulated
RNF19A	0.301154836	0.003986959	Up-regulated
NUDT4	0.295373189	5.97E-03	Up-regulated
RAP1GAP	0.291369544	0.003492746	Up-regulated
GABARAPL1	0.27683293	0.036199859	Up-regulated
GABPB1	0.273490872	0.014147668	Up-regulated
MAPK8	0.266316793	3.03E-04	Up-regulated
TSPAN5	0.264605096	2.71E-02	Up-regulated
SLC25A28	0.256588853	0.000895817	Up-regulated
EPOR	0.255145126	0.0006535	Up-regulated
FBXW7	0.251025813	0.025693917	Up-regulated
TMEM199	-0.251147413	0.013256487	Down-regulated
PRDX1	-0.253606198	0.0000693	Down-regulated
ATP6V1D	-0.259191099	0.022027048	Down-regulated
NFE2	-0.263669448	0.0247544	Down-regulated
SC5D	-0.274069579	0.034760309	Down-regulated
SQSTM1	-0.276564879	0.002722056	Down-regulated
AIFM2	-0.29156169	0.000165819	Down-regulated
BLVRA	-0.300414531	0.001453126	Down-regulated
MCOLN1	-0.305023194	0.035999755	Down-regulated
SLC22A4	-0.305836824	0.033886512	Down-regulated
DNM2	-0.331318789	0.001196319	Down-regulated
HMBS	-0.333975649	0.004073285	Down-regulated
ATP6V1B2	-0.337334019	0.0000799	Down-regulated
FLT3	-0.347612091	0.00974279	Down-regulated
ATP6V1A	-0.355101303	0.0000497	Down-regulated
BLVRB	-0.359047166	4.81E-03	Down-regulated
ATP6V0D1	-0.367305774	0.008571053	Down-regulated
HTATIP2	-0.368213212	0.000397317	Down-regulated
SCD	-0.381577518	0.022027048	Down-regulated
FLVCR2	-0.383264464	0.012512065	Down-regulated
SLC39A8	-0.394817761	0.008463876	Down-regulated
SLC3A2	-0.402939154	0.001056622	Down-regulated
SLC25A37	-0.408231759	0.005071735	Down-regulated
CTNS	-0.422643646	0.0000434	Down-regulated
HSPA5	-0.42445206	0.037190781	Down-regulated

CTSB	-0.443300984	0.001697036	Down-regulated
GPX2	-0.44705746	0.010434244	Down-regulated
GCLC	-0.450491283	0.011174022	Down-regulated
ABCB6	-0.459126647	0.0000119	Down-regulated
MSMO1	-0.459453732	0.047095921	Down-regulated
GCLM	-0.482237143	0.001005209	Down-regulated
UCP2	-0.48583649	0.018825182	Down-regulated
SLC11A1	-0.491441728	0.02763908	Down-regulated
SLC6A9	-0.51332698	0.0000103	Down-regulated
G6PD	-0.51658732	0.0000198	Down-regulated
TLR4	-0.521770415	0.000311202	Down-regulated
SRXN1	-0.524839037	0.000235263	Down-regulated
CAPG	-0.528341976	0.003860613	Down-regulated
STEAP3	-0.534129687	0.000317353	Down-regulated
TBXAS1	-0.544825943	0.000150462	Down-regulated
FADS2	-0.565987196	0.020965623	Down-regulated
AKR1C3	-0.58523225	0.001089038	Down-regulated
CYBB	-0.591425536	0.011401412	Down-regulated
MPP1	-0.61651265	3.62E-04	Down-regulated
NCF2	-0.618504721	0.000335126	Down-regulated
AKR1C2	-0.639234931	0.010024044	Down-regulated
IDH1	-0.711495669	0.0000119	Down-regulated
CBS	-0.791207365	1.07E-05	Down-regulated
PSAT1	-0.797090133	0.006424486	Down-regulated
PGD	-0.837316377	0.000000342	Down-regulated
TXNRD1	-0.838781973	1.67E-08	Down-regulated
LTF	-0.884583255	1.03E-02	Down-regulated
CTSE	-0.995644361	0.001005209	Down-regulated
SLC7A11	-1.023905563	0.003429183	Down-regulated
CYP1B1	-1.190036878	0.004058066	Down-regulated
NQO1	-1.233015109	0.00000259	Down-regulated
HMOX1	-1.449264008	0.0000712	Down-regulated
LCN2	-1.564206439	0.000164052	Down-regulated
