# Supplementary material for: Iron Metabolism and Idiopathic Pulmonary Arterial Hypertension: New Insights from Bioinformatic Analysis
Source: Biomed Res Int. 2021 Oct 22;2021:5669412. doi: 10.1155/2021/5669412 (PMC8556088; doi:10.1155/2021/5669412)
Supplement: Supplementary Materials — are available online at DOI: 10.6084/m9.figshare.14877513. Figure S1: gene expression vioplot of GSE117261 and GSE15197 after normalization. Figure S2: correlation heat map of differentially expressed iron metabolism-related genes in GSE117261. Figure S3: predicted target genes of downregulated miRNA. Figure S4: predicted target genes of upregulated miRNA. Figure S5: key modules identified by the Cytoscape plugin MCODE. Table S1: the merged iron metabolism-related gene set. Figure S6: correlation heat map of immune cells in GSE117261 and GSE15197. Figure S7: linear regression analysis between expression of key genes and the proportion of immune cells in GSE117261 and GSE15197. Figure S8: top 10 targeted drugs predicted in the DSigDB database ranked by FDR. Table S1: the merged iron metabolism related gene set. Table S2: dysregulated miRNAs in IPAH samples. Table S3: differentially expressed iron metabolism-related gene set. Table S4: rank values of differentially expressed iron metabolism-related genes by MCC algorithm. Table S5: the proportion of infiltrating immune cells estimated by the CIBERSORT algorithm in GSE117261. Table S6: the proportion of infiltrating immune cells estimated by the CIBERSORT algorithm in GSE15197. Table S7: predicted target drug using the DSigDB database. [file 5669412.f1.zip › Table S4 Rank values of differentially expressed iron metabolism related genes by MCC algorithm.pdf]

Table S4 Rank values of DEIMRGs

Node_name	Degree
HMOX1	46935
GCLC	46874
NQO1	46744
TXNRD1	46717
GCLM	46370
SRXN1	46081
GPX2	45606
PRDX1	40472
SLC7A11	40447
G6PD	5074
SQSTM1	996
HSPA5	543
JUN	417
MAPK8	292
TLR4	290
VEGFA	265
AKR1C2	240
CYBB	142
AKR1C3	123
CBSL	122
NFE2	64
ALAS2	40
PGD	31
SLC25A37	31
ATP6V1A	28
HEPH	26
ATP6V1B2	26
HBB	26
ATP6V0D1	25
SLC39A8	24
TMEM199	24
ATP6V1D	24
STEAP3	20
CTSB	17
HMBS	17
SLC11A1	16
SLC25A28	16
IDH1	14
HBA2	14
HBD	12
EPOR	11
ABCB6	11
LCN2	9
BLVRB	9
FLT3	8
FBXW7	7
SCD	6
NCF2	6

GABARAPL1	6
PSAT1	6
CYP1B1	5
SLC3A2	4
MSMO1	4
BCL2	4
FLVCR2	4
UCP2	3
CDO1	3
CA2	3
BMP6	3
BTG2	3
BLVRA	3
ABCG2	3
SLC6A9	3
SC5D	2
LTF	2
HERPUD1	2
FADS2	2
DNM2	2
MCOLN1	2
CTNS	2
AIFM2	2
SCARA5	1
TSPAN5	1
IGSF3	1
MPP1	1
ENPP2	1
CTSE	1
NEO1	1
TBXAS1	1
SLC22A4	1
