# Supplementary material for: Iron Metabolism and Idiopathic Pulmonary Arterial Hypertension: New Insights from Bioinformatic Analysis
Source: Biomed Res Int. 2021 Oct 22;2021:5669412. doi: 10.1155/2021/5669412 (PMC8556088; doi:10.1155/2021/5669412)
Supplement: Supplementary Materials — are available online at DOI: 10.6084/m9.figshare.14877513. Figure S1: gene expression vioplot of GSE117261 and GSE15197 after normalization. Figure S2: correlation heat map of differentially expressed iron metabolism-related genes in GSE117261. Figure S3: predicted target genes of downregulated miRNA. Figure S4: predicted target genes of upregulated miRNA. Figure S5: key modules identified by the Cytoscape plugin MCODE. Table S1: the merged iron metabolism-related gene set. Figure S6: correlation heat map of immune cells in GSE117261 and GSE15197. Figure S7: linear regression analysis between expression of key genes and the proportion of immune cells in GSE117261 and GSE15197. Figure S8: top 10 targeted drugs predicted in the DSigDB database ranked by FDR. Table S1: the merged iron metabolism related gene set. Table S2: dysregulated miRNAs in IPAH samples. Table S3: differentially expressed iron metabolism-related gene set. Table S4: rank values of differentially expressed iron metabolism-related genes by MCC algorithm. Table S5: the proportion of infiltrating immune cells estimated by the CIBERSORT algorithm in GSE117261. Table S6: the proportion of infiltrating immune cells estimated by the CIBERSORT algorithm in GSE15197. Table S7: predicted target drug using the DSigDB database. [file 5669412.f1.zip › Table S6 The proportion of infiltrating immune cells estimated by the CIBERSORT algorithm in GSE15197.pdf]

Table S6 The proportion of infiltrating immune cells estimated by the CIBERSORT algorithm in GSE15197

Immune cells	GSM379337	GSM379338	GSM379339	GSM379340	GSM379341	GSM379342	GSM379343	GSM379344	GSM379345	GSM379346	GSM379347
B cells naive	0	0	0	0	0.02270015	0.030815368	0	0	0	0	0
B cells memory	0.041146789	0.020292051	0.010221705	0.042744021	0	0	0.008747415	0.058690119	0.019613846	0.042092374	0.086599109
Plasma cells	0.06320748	0.01696525	0.023476391	0.045502943	0.023143387	0.000823733	0.008918802	0.033053405	0.017694976	0.017533634	0.039776094
T cells CD8	0.169012528	0.152933513	0.127542725	0.150466065	0.154686537	0	0.138282919	0.195458927	0.143553706	0.025436911	0.098637058
T cells CD4 naive	0	0	0	0	0	0	0	0.02376561	0	0	0
T cells CD4 memory resting	0.017914101	0.063793827	0.079701981	0.014225014	0	0.025980062	0.048241242	0	0	0.07297588	0
T cells CD4 memory activated	0.000692532	0.09708879	0.002361099	0.013180197	0.039021939	0.070403056	0.059547764	0.000475298	0.018167805	0.006595958	0
T cells follicular helper	0	0.014640369	0	0.072497464	0.080077057	0.068061661	0.007369105	0.014721433	0.038870602	0.024119365	0.040806967
T cells regulatory (Tregs)	0.001843098	0	0	0	0.015209567	0	0	0.002327764	0	0	0.014179525
T cells gamma delta	0	0	0	0.036307799	0	0	0	0	0	0	0
NK cells resting	0	0	0	0	0	0	0	0	0	0	0
NK cells activated	0.038165093	0.082549164	0.102280645	0.120039901	0.045641753	0.159930294	0.07928233	0.147281119	0.113603095	0.023347761	0.207605273
Monocytes	0.047726436	0.031330619	0.124160358	0	0.044848097	0.010201289	0.05880327	0.024243737	0.025479966	0.0606555	0.035718424
Macrophages M0	0.26505228	0.125820263	0.199873641	0.178964784	0.317701212	0.168712673	0.091806737	0.169272516	0.153679651	0.258503159	0.062354134
Macrophages M1	0.007065693	0.067601474	8.90E-03	0.033207455	0.019600438	0.105968308	0.04429803	0.097828975	0.032349123	0.00337465	0.008464232
Macrophages M2	0.274938436	0.147111726	0.180669402	0.228947103	0.136089722	0.140257526	0.122970432	0.127342134	0.13758194	0.318348898	0.173503654
Dendritic cells resting	0	0	0	0	0	0	0	0	0	0	0
Dendritic cells activated	0	0.017038942	0.030619583	0	0	0	0.000833558	0	0	0.018760541	0.002165721
Mast cells resting	0.048109898	0	0.056408984	0	0	0	0	0	0	0.051241562	0
Mast cells activated	0.00182212	0.127898037	0	0.040735203	0.056398135	0.174600653	0.280250139	0.100522315	0.272457765	0.021507423	0.17752747
Eosinophils	0	0.00238741	0	0	0.032047803	0.011588368	0.030106389	0	0.004340258	0.009543945	0
Neutrophils	0.023303517	0.032548566	0.053781327	0.02318205	0.012834204	0.03265701	0.02054187	0.005016649	0.022607268	0.045962437	0.052662339

Immune cells	GSM379348	GSM379349	GSM379350	GSM379351	GSM379352	GSM379353	GSM379354	GSM379316	GSM379317	GSM379318
B cells naive	0.049325149	0	0	0	0	0.003539908	0	0	0.038976148	0.016951396
B cells memory	0	0.024996504	0.053015981	0.003574747	0.038464394	0	0.017738078	0.053821906	0	0
Plasma cells	0	0.013487128	0.040631619	0.007678078	0.059973531	0	0.033869344	0.036263694	0	0.008021058
T cells CD8	0.021008831	0.335439922	0.060728763	0.085560031	0.10503694	0.082336664	0.06897453	0	0.048203649	0.051608726
T cells CD4 naive	0	0	0	0	0	0	0	0	0	0
T cells CD4 memory resting	0.070872237	0	0.041783181	0.026771998	0.090979219	0.113306774	0.106299747	0.121152021	0.048176742	0.0461393
T cells CD4 memory activated	0.107012005	0.060472351	0.015534842	0.05347252	0	0.018083777	0.002502059	0.011690592	0.034674231	0.130121687
T cells follicular helper	0.019908182	0.006603481	0.018660544	0.057989597	0.016887447	0.001245179	0.021925617	0.031018373	0.035088159	0.009483136
T cells regulatory (Tregs)	0	0	0.005675685	0.007219639	0.001508435	0	0	0.016194607	0.005864001	0
T cells gamma delta	0	0	0	0.008136668	0	0	0	0	0	0
NK cells resting	0	0	0	0	0	0	0	0	0	0.006929751
NK cells activated	0.206336427	0.144263306	0.041156363	0.065081699	0.086913478	0.156890685	0.126376128	0.15610757	0.043678581	0.077554186
Monocytes	0.054237605	0.14079636	0.127426328	0.021465253	0.015484582	0.013793509	0.007750896	0.162954393	0.092390421	0.128908602
Macrophages M0	0	0.021701416	0.176335469	0.139831708	0.127022086	0.296756861	0.09378172	0	0.201957425	0.133170143
Macrophages M1	0.037453564	0.027123951	0.009077019	0.122214012	0.103483796	0.006751954	0.098993108	0.039081356	0.036823999	0
Macrophages M2	0.096910606	0.1362635	0.265336886	0.229617958	0.275828469	0.136088641	0.173791162	0.166263396	0.259690147	0.120022626
Dendritic cells resting	0	0	0	0.000365659	0	0	0	0	0	0
Dendritic cells activated	0.019212402	0	0	0	0	0.009263307	0.016199447	0.003923485	0	0.110175077
Mast cells resting	0	0	0.054843748	0.13980885	0.013869456	0	0.067987826	0.04490193	0.091597171	0.04070177
Mast cells activated	0.256880125	0.082637894	0.046766646	0	0.040162912	0.134857425	0.040934123	0.081071254	0	0.034246912
Eosinophils	0.033148907	0	0.017258874	0	0	0.011212245	0.062982759	0.012756232	0.018042729	0.013675541
Neutrophils	0.027693959	0.006214187	0.025768052	0.031211584	0.024385256	0.01587307	0.059893458	0.062799191	0.044836596	0.072290089

Immune cells	GSM379319	GSM379320	GSM379321	GSM379322	GSM379323	GSM379324	GSM379325	GSM379326	GSM379327	GSM379328
B cells naive	0.013331539	0.111032663	0	0	0	0	0.00630155	0	0	0.054194754
B cells memory	0	0	0.023921748	0.054120985	0	0	0	0.009505298	0	0
Plasma cells	0.009913073	0.109312166	0.014930751	0.045191006	0.003506919	0.009938383	0	0.028596125	0.036375553	0
T cells CD8	0.09530086	0.071787014	0.113659702	0.152922999	0.043308902	0.045150696	0.066689682	0.036032898	0.151308906	0.046298219
T cells CD4 naive	0	0	0	0	0	0	0	0	0	0
T cells CD4 memory resting	0.178168441	0.07460059	0.019060472	0	0.108188003	0.064803288	0.00948963	0.097248667	0.005882107	0
T cells CD4 memory activated	0	0.084892971	0	0.079481523	0	0.067910856	0	0.013453919	0.0944801	0.046336652
T cells follicular helper	0	0.047810328	0.093925235	0.077405102	0.031229032	0.002215048	0.023955266	0.010467213	0.032341794	0.054921544
T cells regulatory (Tregs)	0	0.01185658	0.003134838	0.007500764	0	0.013271717	0	0	0.018829239	0.032475387
T cells gamma delta	0	0.000394644	0	0	0	0	0	0	0	0
NK cells resting	0	0	0	0	0.019770321	0	0.029720703	0	0	0
NK cells activated	0.209352494	0.049573584	0.122832192	0.012455673	0.020117762	0.072327714	0.010742385	0.035707701	0.092135808	0.036232159
Monocytes	0.062713237	0	0.043540981	0.040416792	0.04094077	0.063308393	0.118387798	0.252650601	0.033941392	0.051193947
Macrophages M0	0.026674842	0.105123485	0.160228124	0.199511258	0.216658704	0.151065189	0.368962328	0.139154918	0.061454948	0.295910716
Macrophages M1	0.073449203	0.007458363	0.074635047	0.027126264	0.017566975	0.063982078	0.00667655	0	0.06716016	0.058658118
Macrophages M2	0.126120671	0.185048333	0.144007624	0.167525156	0.266892092	0.177945564	0.137497246	0.125532399	0.161608467	0.155792581
Dendritic cells resting	0	0	0	0	0	0	0	0	0.026219528	0
Dendritic cells activated	0	0.01656402	0	0.00987719	0.013939539	0	0	0.034036424	0	0
Mast cells resting	0.107387232	0.082372105	0	0	0.076873754	0.174754659	0.035040588	0.108703685	0.162078507	0.085289276
Mast cells activated	0.022640779	0.026844099	0.114533476	0.086823321	0	0.020833729	0	0	0	0
Eosinophils	0.018918755	0.001946097	0.063639719	0.015425617	0.067230084	0.020839306	0.011433214	0.016442158	0.019928768	0
Neutrophils	0.056028875	0.013382958	0.007950091	0.024216352	0.073777142	0.051653381	0.175103059	0.092467994	0.036254723	0.082696646
