# Supplementary material for: Iron Metabolism and Idiopathic Pulmonary Arterial Hypertension: New Insights from Bioinformatic Analysis
Source: Biomed Res Int. 2021 Oct 22;2021:5669412. doi: 10.1155/2021/5669412 (PMC8556088; doi:10.1155/2021/5669412)
Supplement: Supplementary Materials — are available online at DOI: 10.6084/m9.figshare.14877513. Figure S1: gene expression vioplot of GSE117261 and GSE15197 after normalization. Figure S2: correlation heat map of differentially expressed iron metabolism-related genes in GSE117261. Figure S3: predicted target genes of downregulated miRNA. Figure S4: predicted target genes of upregulated miRNA. Figure S5: key modules identified by the Cytoscape plugin MCODE. Table S1: the merged iron metabolism-related gene set. Figure S6: correlation heat map of immune cells in GSE117261 and GSE15197. Figure S7: linear regression analysis between expression of key genes and the proportion of immune cells in GSE117261 and GSE15197. Figure S8: top 10 targeted drugs predicted in the DSigDB database ranked by FDR. Table S1: the merged iron metabolism related gene set. Table S2: dysregulated miRNAs in IPAH samples. Table S3: differentially expressed iron metabolism-related gene set. Table S4: rank values of differentially expressed iron metabolism-related genes by MCC algorithm. Table S5: the proportion of infiltrating immune cells estimated by the CIBERSORT algorithm in GSE117261. Table S6: the proportion of infiltrating immune cells estimated by the CIBERSORT algorithm in GSE15197. Table S7: predicted target drug using the DSigDB database. [file 5669412.f1.zip › Table S7 Predicted target drug using the DSigDB database.pdf]

Table S7 Predicted target drug using the DSigDB database

Term	P-value	Odds Ratio	Combined Score	Genes
celastrol CTD 00002186	6.51E-11	512.783505	12027.55685	TXNRD1;BCL2;MSMO1;SLC7A11;GCLM
cinnamaldehyde CTD 00000671	7.45E-10	309.890625	6513.108816	SRXN1;TXNRD1;BCL2;SLC7A11;GCLM
Diquat dication CTD 00005858	7.78E-10	648.845529	13608.70128	SRXN1;TXNRD1;SLC7A11;GCLM
7-ACA CTD 00001461	9.54E-09	1362.40909	25160.78662	TXNRD1;SLC7A11;GCLM
gedunin CTD 00003449	1.27E-08	312.282353	5678.058245	TXNRD1;MSMO1;SLC7A11;GCLM
EPICHLOROHYDRIN CTD 00005906	2.14E-08	998.9	17641.76329	TXNRD1;SLC7A11;GCLM
CHLOROPICRIN CTD 00003338	2.14E-08	998.9	17641.76329	TXNRD1;SLC7A11;GCLM
securinine MCF7 UP	3.48E-08	832.291667	14293.03881	TXNRD1;SLC7A11;GCLM
oxyphenbutazone MCF7 UP	4.03E-08	788.447368	13424.62308	TXNRD1;SLC7A11;GCLM
carmustine MCF7 UP	4.64E-08	748.9875	12648.18423	TXNRD1;SLC7A11;GCLM
HEXANE CTD 00001239	0.000000053	713.285714	11950.14616	SRXN1;TXNRD1;GCLM
5194442 MCF7 UP	6.02E-08	680.829546	11319.45464	TXNRD1;SLC7A11;GCLM
ebselen MCF7 UP	0.000000068	651.195652	10747.0207	TXNRD1;SLC7A11;GCLM
benzoic acid CTD 00007316	7.65E-08	624.03125	10225.30468	SRXN1;TXNRD1;GCLM
lactic acid CTD 00007283	7.65E-08	624.03125	10225.30468	SRXN1;TXNRD1;GCLM
isotretinoin MCF7 UP	8.57E-08	599.04	9748.001391	TXNRD1;SLC7A11;GCLM
methyl salicylate CTD 00001586	0.000000118	534.776786	8533.330719	SRXN1;TXNRD1;GCLM
nifuroxazide MCF7 UP	0.000000118	534.776786	8533.330719	TXNRD1;SLC7A11;GCLM
1,6-Diisocyanatohexane CTD 00000793	0.00000013	516.310345	8187.916393	TXNRD1;SLC7A11;GCLM
alpha-Hexylcinnamaldehyde CTD 00002937	0.000000143	499.075	7867.096685	SRXN1;TXNRD1;GCLM
isopropanol CTD 00007325	0.000000143	499.075	7867.096685	SRXN1;TXNRD1;GCLM
cinnamyl alcohol CTD 00001006	0.000000187	453.636364	7029.00711	SRXN1;TXNRD1;GCLM
semustine MCF7 UP	0.000000203	440.272059	6784.76713	TXNRD1;SLC7A11;GCLM
Ammonium hexachloroplatinate(IV) CTD 00000945	0.000000239	415.770833	6339.845805	TXNRD1;SLC7A11;GCLM
Bandrowski's base CTD 00002216	0.000000258	404.513514	6136.713336	SRXN1;TXNRD1;GCLM
tert-Butylhydroquinone CTD 00000961	0.0000003	383.730769	5763.945414	TXNRD1;BCL2;GCLM
5182598 MCF7 UP	0.000000322	374.11875	5592.564435	TXNRD1;SLC7A11;GCLM
securinine HL60 UP	0.000000346	364.97561	5430.167744	TXNRD1;SLC7A11;GCLM
hydrogen peroxide CTD 00006118	0.000000756	121289	1709522.752	SRXN1;TXNRD1;BCL2;TSPAN5;MSMO1;SLC7A11;GCLM
Tetradioxin CTD 00006848	0.00000839	113624	1328140.359	SRXN1;TXNRD1;BCL2;TSPAN5;MSMO1;SLC7A11;GCLM
mechlorethamine CTD 00006251	0.000016	499.425	5514.422774	TXNRD1;BCL2
estradiol CTD 00005920	0.0000224	109648	1173815.852	SRXN1;TXNRD1;BCL2;TSPAN5;MSMO1;SLC7A11;GCLM
benzo[a]pyrene CTD 00005488	0.0000258	109032	1151876.014	SRXN1;TXNRD1;BCL2;TSPAN5;MSMO1;SLC7A11;GCLM
VALPROIC ACID CTD 00006977	0.002138353	81816	502981.7918	SRXN1;TXNRD1;BCL2;TSPAN5;MSMO1;SLC7A11;GCLM
