# Supplementary material for: Iron Metabolism and Idiopathic Pulmonary Arterial Hypertension: New Insights from Bioinformatic Analysis
Source: Biomed Res Int. 2021 Oct 22;2021:5669412. doi: 10.1155/2021/5669412 (PMC8556088; doi:10.1155/2021/5669412)
Supplement: Supplementary Materials — are available online at DOI: 10.6084/m9.figshare.14877513. Figure S1: gene expression vioplot of GSE117261 and GSE15197 after normalization. Figure S2: correlation heat map of differentially expressed iron metabolism-related genes in GSE117261. Figure S3: predicted target genes of downregulated miRNA. Figure S4: predicted target genes of upregulated miRNA. Figure S5: key modules identified by the Cytoscape plugin MCODE. Table S1: the merged iron metabolism-related gene set. Figure S6: correlation heat map of immune cells in GSE117261 and GSE15197. Figure S7: linear regression analysis between expression of key genes and the proportion of immune cells in GSE117261 and GSE15197. Figure S8: top 10 targeted drugs predicted in the DSigDB database ranked by FDR. Table S1: the merged iron metabolism related gene set. Table S2: dysregulated miRNAs in IPAH samples. Table S3: differentially expressed iron metabolism-related gene set. Table S4: rank values of differentially expressed iron metabolism-related genes by MCC algorithm. Table S5: the proportion of infiltrating immune cells estimated by the CIBERSORT algorithm in GSE117261. Table S6: the proportion of infiltrating immune cells estimated by the CIBERSORT algorithm in GSE15197. Table S7: predicted target drug using the DSigDB database. [file Table S5 The proportion of infiltrating immune cells estimated by the CIBERSORT algorithm in GSE117261 (1).pdf › Table S5 The proportion of infiltrating immune cells estimated by the CIBERSORT algorithm in GSE117261 (1).pdf]

Table S5 The proportion of infiltrating immune cells estimated by the CIBERSORT algorithm in GSE117261

Immune cells	GSM3290067	GSM3290068	GSM3290069	GSM3290070	GSM3290072	GSM3290074	GSM3290075	GSM3290077	GSM3290082	GSM3290084	GSM3290089
B cells naive	0.011399143	0.007727291	0.003251831	0.010996847	0.041722218	0.019910032	0.025751408	0.005376494	0	0.011495072	0.015694457
B cells memory	0	0.019144535	0.006052948	0.014632819	0	0.041980225	0	0	0.036343911	0.003994844	0.01984919
Plasma cells	0.024882827	0.023677563	0.025385552	0.026209099	0.014816619	0.010333572	0.024316346	0.018884722	0.0149057	0.015609438	0.03036078
T cells CD8	0.054787837	0.050297679	0.041531194	0.092480767	0.100688224	0.056419322	0.014926629	0.034992107	0.026674472	0.05727	0.128589235
T cells CD4 naive	0	0	0	0	0	0	0	0	0	0	0
T cells CD4 memory resting	0.221967769	0.305712012	0.096879125	0.182144938	0.165131882	0.198374736	0.182183443	0.144541088	0.134330266	0.214400479	0.330337344
T cells CD4 memory activated	0.012222283	0.020904073	0.00637843	0.003577452	0.006437973	0.002216852	0.000549087	0.007578748	0	0.00213368	0
T cells follicular helper	0	0	0	0	0	0	0	0	0	0	0
T cells regulatory (Tregs)	0	0	0	0	0	0	0	0	0	0	0
T cells gamma delta	0.010651896	0	0	0	0	0.094462256	0	0.045694771	0.020440257	0	0
NK cells resting	0.007441237	0.04726042	0.03573616	0.000184188	0.04572014	0.010467923	0	0.027366682	0.057134271	0	0.009510363
NK cells activated	0	0	0.000382211	0.007660779	0	0	0.003486885	0	0	0.025139196	0
Monocytes	0.033303461	0.057049439	0.065947417	0.024095014	0.075791587	0.086397456	0.02088072	0.036983165	0.016601375	0.04695402	0.041048733
Macrophages M0	0.031336258	0.015526346	0.262003044	0.181370998	0	0	0.070032352	0.161489874	0.129309502	0	0
Macrophages M1	0.053866323	0	0.003383135	0.004085762	0.016785694	0.061982683	0	0.005747243	0	0	0
Macrophages M2	0.370503771	0.263219138	0.237021264	0.241322737	0.295801575	0.209402771	0.225240694	0.168858601	0.30975177	0.246759888	0.197377708
Dendritic cells resting	0	0	0	0	0	0.007921812	0	0	0	0	0
Dendritic cells activated	0.007682035	0.043247539	0.062790257	0.024491452	0.085321372	0.016231443	0.091767571	0.113384014	0.142370709	0.184852387	0.081162726
Mast cells resting	0.111961289	0.146075219	0.136568296	0.154949894	0.086034232	0.118345823	0.335255466	0.128384044	0.082825604	0.157904594	0.126443104
Mast cells activated	0	0	0	0	0	0	0	0	0	0	0
Eosinophils	0.00019594	0.000158748	0	0.001856018	0	0	0	0	0.008407761	0.008115527	0.007811019
Neutrophils	0.047797932	0	0.016689133	0.029941236	0.065748485	0.065553094	0.005609398	0.100718445	0.020904403	0.025370875	0.011815341

Immune cells	GSM3290095	GSM3290099	GSM3290103	GSM3290105	GSM3290106	GSM3290107	GSM3290108	GSM3290109	GSM3290113	GSM3290116	GSM3290126
B cells naive	0.020577105	0.019589112	0.020985608	0.004260627	0.009024951	0.009393958	0.009574697	9.19E-05	0	0.020551419	0.033072048
B cells memory	0.000302713	0.017890654	0	0.001763361	0.001824141	0.004948831	0	0	0.093308802	0.023299743	0
Plasma cells	0.035530546	0.042904244	0.020937722	0.02218102	0.019069236	0.019504121	0.063596191	0.00766825	0.090214903	0.026517867	0.042848921
T cells CD8	0	0.037849866	0.019379913	0.023555043	0.035549048	0.036944854	0.048117775	0.352639967	0.139471697	0.076090008	0.006037474
T cells CD4 naive	0	0	0	0	0	0	0	0	0	0	0
T cells CD4 memory resting	0.220891607	0.240322489	0.193243787	0.273819253	0.163878542	0.243716862	0.22875288	0.085528751	0.116910974	0.219322475	0.242577704
T cells CD4 memory activated	0.009916698	0	0	0.016479481	0.004240778	0	0	0.015003667	0.01892633	0	0
T cells follicular helper	0	0.023429409	0	0	0	0.012200345	0	0	0	0.036462818	0
T cells regulatory (Tregs)	0	0	0	0	0	0	0	0	0	0	0
T cells gamma delta	0.083375219	0	0	0.015777537	0	0	0.041786021	0.17922625	0.082148292	0	0.048499232
NK cells resting	0	0.062260684	0.032319137	0.009742679	0.050816167	0.006697384	0.002394446	0	0	0	0.015644199
NK cells activated	0	0.040517279	0	0	0	0.050053882	0	0	0	0.004682715	0
Monocytes	0.029406314	0.05319436	0.027267882	0.028947199	0.030264947	0.055705076	0.014990462	0	0	0.04331712	0.00028932
Macrophages M0	0.020564897	0.016298937	0.033313789	0.062075925	0	0	0.07281872	0.070157272	0.125284596	0.074486136	0.075997895
Macrophages M1	0	0.015332851	0	0.007926735	0	0.053049256	0.023696455	0.078044727	0.065735402	0.027082649	0.05980009
Macrophages M2	0.191210052	0.163428648	0.295223532	0.273430685	0.329846374	0.272209059	0.269960436	0.161192827	0.158264001	0.163199831	0.203757871
Dendritic cells resting	0	0	0	0	0	0	0	0.004574967	0	0	0
Dendritic cells activated	0.109540628	0.132414033	0.121088028	0.033635339	0.105116769	0.053053977	0.057692931	0	0.04389716	0.104004647	0.058941722
Mast cells resting	0.186514844	0.083368368	0.2015552	0.107336915	0.229495197	0.165979243	0.121503061	0.040799962	0.052833323	0.172094072	0.12595408
Mast cells activated	0	0	0	0	0	0	0	0	0	0	0
Eosinophils	0.003589386	0.00336968	0	0	0	0	0.001722301	0	0	0	0
Neutrophils	0.08857999	0.047829386	0.034685401	0.119068201	0.020873851	0.016543152	0.043393622	0.005071507	0.013004519	0.008888501	0.086579443

Immune cells	GSM3290127	GSM3290128	GSM3290129	GSM3290136	GSM3290137	GSM3290138	GSM3290140	GSM3290143	GSM3290145	GSM3290148	GSM3290083
B cells naive	0.013762826	0	0	0.011478389	0.01376173	0.028294785	0.015749056	0.021260796	0	0.016561097	0.004521733
B cells memory	0	0.102264912	0.006572129	0.00092065	0.00526974	0	0.017362567	0	0.00485198	0.005354707	0.038944794
Plasma cells	0.02090729	0.017079169	0.014277303	0.028573628	0.021564137	0.018899134	0.025303904	0.017094304	0.009771361	0.030883118	0.005005584
T cells CD8	0	0.010273024	0.038744735	0.058508625	0.074208242	0.031721092	0.054321904	0.021161594	0.012768284	0.081799008	0.011333253
T cells CD4 naive	0	0	0	0	0	0	0	0	0	0	0.011535452
T cells CD4 memory resting	0.14181251	0.12498028	0.153042236	0.287886617	0.258559917	0.309622061	0.330822107	0.1523256	0.14103091	0.215373382	0.154088171
T cells CD4 memory activated	0.003647625	0.008595283	0	0	0	0	0	0	0.000888071	0	0.042198104
T cells follicular helper	0	0.004175314	0	0	0	0	0	0	0	0	0
T cells regulatory (Tregs)	0	0	0	0	0	0	0	0	0	0	0
T cells gamma delta	0	0.042473005	0.015644745	0	0	0	0	0	0.005849878	0	0.016283245
NK cells resting	0.045984589	0	0.027940558	0.015815935	0.016318288	0.017256213	5.14E-05	0.012601859	0.039628953	0.016864533	0.043247483
NK cells activated	0	0.003004645	0	0	0	0.028197957	0	0	0	0	0
Monocytes	0.128608422	0.005480368	0.027068978	0.063946995	0.042006335	0.049482728	0.02920792	0.012296103	0.173307322	0.064654329	0.255454747
Macrophages M0	0.042068714	0.010641846	0.06945128	0	0	0.005204053	0	0.125163923	0.111988133	0.110866396	0.045016104
Macrophages M1	0	0.02436908	0.009783362	0	0.001310248	0.06367324	0.031162552	0.019370365	0.013646979	0	0.088815096
Macrophages M2	0.133605306	0.231880295	0.263947901	0.250736461	0.228526405	0.248033187	0.222407045	0.321959869	0.232851448	0.237249539	0.164422169
Dendritic cells resting	0	0	0	0	0	0	0.003430406	0	0	0	0
Dendritic cells activated	0.257272457	0.083071434	0.111597976	0.039315743	0.038132807	0.029463975	0.017670594	0.150002529	0.076484135	0.046693832	0.002082317
Mast cells resting	0.138620899	0.32626446	0.215067545	0.179011281	0.265532157	0.120190674	0.24548776	0.092538736	0.079695074	0.145446646	0.015627912
Mast cells activated	0	0	0	0	0	0	0	0	0	0	0
Eosinophils	0	0.005446884	0	0.005545576	0	0	0	0.008069604	0	0.005514521	0
Neutrophils	0.073709361	0	0.046861252	0.058260101	0.034809993	0.049960902	0.00702274	0.046154719	0.097237471	0.022738893	0.101423836

Immune cells	GSM3290085	GSM3290086	GSM3290088	GSM3290091	GSM3290092	GSM3290093	GSM3290094	GSM3290097	GSM3290098	GSM3290100	GSM3290101
B cells naive	0.013152524	0.062381844	0	0.018384308	0.014795283	0.015823725	0.001365087	0	0.031034375	0.020131312	0.00935071
B cells memory	0	0	0.019701744	0	0	0	0.006994936	0.001905558	0	0	0
Plasma cells	0.024593568	0.027827939	0.010549171	0.043677127	0.021426504	0.03655747	0.028135601	0.050120937	0.014639498	0.015939973	0.021960929
T cells CD8	0.058376092	0.03111655	0.05013883	0.016098219	0.0203395	0.011642321	0.016703637	0.009937108	0.023471014	0.02911122	0.009882779
T cells CD4 naive	0.040753604	0	0	0	0	0	0	0	0.004566084	0.067142589	0
T cells CD4 memory resting	0.257293941	0.161068037	0.075493169	0.181962831	0.140520273	0.162165623	0.167241668	0.21452755	0.115517478	0.150746602	0.193236247
T cells CD4 memory activated	0	0	0.011979459	0	0.009393904	0	0	0	0	0.019175972	0
T cells follicular helper	0	0	0	0	0	0	0	0	0	0	0
T cells regulatory (Tregs)	0	0	0	0	0	0	0	0	0	0	0
T cells gamma delta	0	0	0	0	0	0	0	0	0	0	0
NK cells resting	0	0.064369572	0.021503588	0.016044843	0.043461589	0.027272632	2.85E-05	0.025421498	0.019517088	0.027312917	0
NK cells activated	0	0	0	0	0	0	0.016958419	0.008672261	0.004974158	0	0.013018269
Monocytes	0.137669764	0.090306258	0.029458991	0.029572138	0.111962407	0.136451525	0	0.059222707	0.097436655	0.109362997	0.140090827
Macrophages M0	0	0.095534809	0.247874431	0.097859553	0.11229624	0.075770204	0.121011648	0.070282155	0.17965127	0.064388217	0.08222636
Macrophages M1	0	0	0	0	0.004675275	0.000517836	0.000109654	0	0	0	0.000139066
Macrophages M2	0.244595	0.208699169	0.258910514	0.229253594	0.32726465	0.311939959	0.258340126	0.124710337	0.081742236	0.236876604	0.20867737
Dendritic cells resting	0	0	0	0	0	0	0	0	0	0	0
Dendritic cells activated	0.024856973	0.082495434	0.073634704	0.088138544	0.02005358	0.020032203	0.080758959	0.165947089	0.216456557	0.014804689	0.077075106
Mast cells resting	0.141601048	0.025132099	0.012384601	0.163896445	0.093495821	0.134452475	0.157754896	0.140377586	0.038647925	0.091880827	0.153984396
Mast cells activated	0	0.021833353	0.043514228	0	0	0	0	0	0	0	0
Eosinophils	0	0.015645976	0.007352649	0	0	0	0	0.022357994	0.009942952	0	0
Neutrophils	0.057107487	0.11358896	0.137503922	0.115112399	0.080314973	0.067374026	0.144596822	0.106517219	0.162402711	0.15312608	0.090357939

Immune cells	GSM3290102	GSM3290118	GSM3290119	GSM3290122	GSM3290123	GSM3290124	GSM3290125	GSM3290131	GSM3290132	GSM3290133	GSM3290134	GSM3290146	GSM3290147
B cells naive	0	0.011862819	0.025517164	0.011170006	0.032845012	0.022327796	0.013741063	0.012800361	0	0	0.012013725	0.031209313	0
B cells memory	0.022539728	0.011333353	0	0.011386309	0	0	0.007156331	0.001546991	0.002704601	0.016245556	0.006259923	0.039510359	0.010502593
Plasma cells	0.013990286	0.017629862	0.018650006	0.023134239	0.01586039	0.036737084	0.018280845	0.018469537	0.018062789	0.015165011	0.017987811	0.044128029	0.011857514
T cells CD8	0.02958417	0.047939486	0.017586347	0.05682255	0.015601645	0.007627379	0.054314489	0.029218295	0.02084339	0.036551828	0.074439285	0.029576211	0.027590201
T cells CD4 naive	0	0	0.054782606	0.049513399	0	0	0.004605095	0.045057914	0	0	0	0	0
T cells CD4 memory resting	0.096611279	0.202477322	0.171598991	0.185806263	0.125476205	0.098766482	0.210474222	0.199663769	0.097706588	0.161505267	0.126726966	0.09509335	0.10917553
T cells CD4 memory activated	0	0.002741617	0.012761019	0	0	0.00025539	0.014444423	0	0	0	0.004296841	4.26E-05	0
T cells follicular helper	0.000340036	0	0	0	0	0	0	0	0	0	0	0	0
T cells regulatory (Tregs)	0	0	0	0	0	0	0	0	0	0	0	0	0
T cells gamma delta	0	0	0	0	0	0	0	0	0.003486124	0	0	0	0
NK cells resting	0.023113457	0.014379642	0.049256325	0.0637215	0.038744055	0.029648124	0.040531871	0.024056297	0.056434141	0.027618443	0.012241982	0.016829179	0.047358753
NK cells activated	0.001185925	0.023246705	0	0	0	0.002104901	0	0.016452221	0	0.007657989	0.016743128	0	0
Monocytes	0.102116864	0.050456501	0.234924999	0.073176967	0.212458839	0.148994132	0.06224087	0.088760456	0.171880701	0.045927004	0.156400714	0	0.157394414
Macrophages M0	0.101735213	0.235696192	0	0.048822045	0.061174741	0.198696561	0.120594658	0.166429241	0.059100698	0.223894994	0.006878253	0.224355521	0.032678633
Macrophages M1	0.002383761	0.004246496	0.002289526	0	0	0.009048883	0	0	0.00223713	0	0.005469484	0	0.030983132
Macrophages M2	0.206108516	0.198147981	0.127701018	0.271713744	0.150805188	0.237887543	0.210100297	0.125404845	0.187067537	0.188832542	0.221106243	0.302893889	0.291642814
Dendritic cells resting	0	0.0108375	0	0.002145359	0	0	0.002611114	0	0	0	0	0	0
Dendritic cells activated	0.157434319	0.016877752	0.008188653	0.022658885	0.024251621	0	0.044666039	0.031805438	0.152356938	0.075312054	0.033686355	0.001707688	0.124159857
Mast cells resting	0.142235686	0.115990475	0.090489546	0.08514832	0	0.105869427	0.118942912	0.174650545	0.081242974	0.125161151	0.206655509	0.12443553	0.106048031
Mast cells activated	0	0	0	0	0.078135413	0	0	0	0	0	0	0	0
Eosinophils	0	0	0	0	0	0	0	0	0	0	0	0	0
Neutrophils	0.100620758	0.036136299	0.1862538	0.094780413	0.24464689	0.102036299	0.07729577	0.06568409	0.146876389	0.076128162	0.099093782	0.090218363	0.050608527
